# Native lamin A/C proteomes and novel partners from heart and skeletal muscle in a mouse chronic inflammation model of human frailty

**DOI:** 10.3389/fcell.2023.1240285

**Published:** 2023-10-23

**Authors:** Fatima D. Elzamzami, Arushi Samal, Adith S. Arun, Tejas Dharmaraj, Neeti R. Prasad, Alex Rendon-Jonguitud, Lauren DeVine, Jeremy D. Walston, Robert N. Cole, Katherine L. Wilson

**Affiliations:** ^1^ Department of Cell Biology, Johns Hopkins University School of Medicine, Baltimore, MD, United States; ^2^ Department of Biological Chemistry, Johns Hopkins University School of Medicine, Baltimore, MD, United States; ^3^ Division of Geriatric Medicine and Gerontology, Johns Hopkins University School of Medicine, Baltimore, MD, United States

**Keywords:** lamin A, Emery-Dreifuss muscular dystrophy, Fam210a, Perm1, AldoA, Lmcd1, Tmem38a, Phf2

## Abstract

Clinical frailty affects ∼10% of people over age 65 and is studied in a chronically inflamed (Interleukin-10 knockout; “IL10-KO”) mouse model. Frailty phenotypes overlap the spectrum of diseases (“laminopathies”) caused by mutations in *LMNA*. *LMNA* encodes nuclear intermediate filament proteins lamin A and lamin C (“lamin A/C”), important for tissue-specific signaling, metabolism and chromatin regulation. We hypothesized that wildtype lamin A/C associations with tissue-specific partners are perturbed by chronic inflammation, potentially contributing to dysfunction in frailty. To test this idea we immunoprecipitated native lamin A/C and associated proteins from skeletal muscle, hearts and brains of old (21–22 months) IL10-KO versus control C57Bl/6 female mice, and labeled with Tandem Mass Tags for identification and quantitation by mass spectrometry. We identified 502 candidate lamin-binding proteins from skeletal muscle, and 340 from heart, including 62 proteins identified in both tissues. Candidates included frailty phenotype-relevant proteins Perm1 and Fam210a, and nuclear membrane protein Tmem38a, required for muscle-specific genome organization. These and most other candidates were unaffected by IL10-KO, but still important as potential lamin A/C-binding proteins in native heart or muscle. A subset of candidates (21 in skeletal muscle, 30 in heart) showed significantly different lamin A/C-association in an IL10-KO tissue (*p* < 0.05), including AldoA and Gins3 affected in heart, and Lmcd1 and Fabp4 affected in skeletal muscle. To screen for binding, eleven candidates plus prelamin A and emerin controls were arrayed as synthetic 20-mer peptides (7-residue stagger) and incubated with recombinant purified lamin A “tail” residues 385–646 under relatively stringent conditions. We detected strong lamin A binding to peptides solvent exposed in Lmcd1, AldoA, Perm1, and Tmem38a, and plausible binding to Csrp3 (muscle LIM protein). These results validated both proteomes as sources for native lamin A/C-binding proteins in heart and muscle, identified four candidate genes for Emery-Dreifuss muscular dystrophy (CSRP3, LMCD1, ALDOA, and PERM1), support a lamin A-interactive molecular role for Tmem38A, and supported the hypothesis that lamin A/C interactions with at least two partners (AldoA in heart, transcription factor Lmcd1 in muscle) are altered in the IL10-KO model of frailty.

## Introduction

Clinical frailty affects ∼10% of people over age 65 and associates with disproportionately high rates of morbidity and mortality (Morley et al., 2013). Diagnosis is based on a spectrum of phenotypes, measured by a Frailty Index score, that can include reduced hand-grip strength, slowed walking, exercise intolerance, unexplained weight loss, reduced cognition or other diagnostic features (Lewsey et al., 2020; Xue et al., 2020). Because clinical frailty also strongly correlates with chronic inflammation, it is studied in a C57Bl/6 mouse model genetically deficient for the anti-inflammatory cytokine, Interleukin-10 (IL-10 B6.129P2-*IL 10* ^*tm/tm*^/J mice; Walston et al., 2008). These *IL10* ^*tm/tm*^ (henceforth ‘IL10-KO’) mice experience lifelong chronic inflammation and exhibit multiple phenotypes consistent with human frailty including increased expression of NF-kB-dependent inflammatory mediators (e.g., IL-1β, TNFα, IFγ, IL-6, chemokine ligand-1; Walston et al., 2008; Ma et al., 2021) as well as age-associated reductions in strength, altered skeletal muscle gene expression, altered insulin signaling (high IGF-1), impaired mitochondrial degradation, high blood pressure, vascular stiffness, reduced fat, endothelial dysfunction, dysregulated tyrosine degradation and higher mortality (Sikka et al., 2013; Westbrook et al., 2017; Lewsey et al., 2020; Malinina et al., 2020; Westbrook et al., 2020). Though primarily a model for chronic inflammation, IL10-KO mice are valuable for many purposes including the study of frailty. There may be other pathways that also lead to frailty that were not investigated here.

Muscle weakening and metabolic disorders are also characteristic of the spectrum of diseases caused by mutations in *LMNA* (“laminopathies”; Charar and Gruenbaum, 2017; Brull et al., 2018; Kreienkamp and Gonzalo, 2020). *LMNA* encodes two abundant nuclear intermediate filament proteins named lamin A and lamin C. Each self-polymerizes, forming lamin A filaments and lamin C filaments in the nucleus. These filaments and two others (lamin B1 and lamin B2) interact with nuclear membrane proteins and chromatin to form nuclear “lamina” networks collectively responsible for nuclear structure, genome integrity, tissue-specific 3D genome organization and tissue-specific gene regulation (Simon and Wilson, 2011; de Las Heras et al., 2013; Zuleger et al., 2013; Harr et al., 2015; van Steensel and Belmont, 2017). Mutations in *LMNA* cause over 15 genetically-dominant disorders including Emery-Dreifuss muscular dystrophy, cardiomyopathy, lipodystrophy, neuropathy, insulin resistance, “accelerated aging” disorders (e.g., Hutchinson-Gilford Progeria (Brull et al., 2018) and juvenile idiopathic inflammatory myopathy (Moraitis et al., 2015); see the *Universal Mutations Database* (http://www.umd.be/LMNA/). *LMNA* mutations are also reported in ∼11% of ‘metabolic syndrome’ patients (Decaudain et al., 2007; Dutour et al., 2011; Desgrouas et al., 2020), 10% of dilated cardiomyopathy patients (Captur et al., 2018) and 27% of heart transplant patients (Kayvanpour et al., 2017).

*LMNA* missense mutations are too rare (less than 1%) in large populations (Florwick et al., 2017) to account for the staggering prevalence of clinical frailty. However, given their upstream roles in signaling and gene regulation, anything that perturbs lamins or their interactions has the potential to disrupt tissue-specific functions, especially in striated muscle. For example, reduced expression of lamin A/C protein associates with osteosarcopenia in human frailty, as measured in circulating osteoprogenitor cells (Al Saedi et al., 2018); similarly, reduced lamin A/C expression in mice due to haploinsufficiency correlates with reduced anabolic response to exercise (Duque et al., 2011). Conversely, increased expression of lamin A/C mRNA and protein, seen in adipose tissue macrophages, is proposed to contribute to obesity-induced insulin resistance by affecting NF-kB signaling in myeloid cells (Kim et al., 2018). Inflammatory signaling is mediated by phosphorylation or *O*-GlcNAcylation of many proteins (Hart, 2014; Li et al., 2019), with the potential to influence lamins A/C (Simon et al., 2018; Lin et al., 2020; Zheng et al., 2022) or partners important for tissue-specific signaling, genome organization or gene expression (Worman and Schirmer, 2015; Wong et al., 2021a). The mechanisms of laminopathies and their hypothetical relationship to human frailty mechanisms are largely unknown due to a lack of knowledge about lamin-dependent proteins in affected tissues such as heart.

We hypothesized that chronic inflammation alters lamin A/C interactions with frailty-relevant partners in muscle, heart or brain. Native lamin A/C proteomes have been reported to our knowledge in only two tissues: *postmortem* human muscle and adipose (Bar et al., 2018). A pioneering biochemical strategy to purify and identify nuclear membrane proteins from native tissues yielded hundreds of novel proteins (Schirmer et al., 2003; Wong et al., 2014), with the potential to bind A- or B-type lamins. Most knowledge about lamin A/C proteomes is based on three approaches: a) proximity labeling (Bosch et al., 2021) in engineered cultured cells such as HEK293 cells (Roux et al., 2012; Go et al., 2021) or fibroblasts (Xie et al., 2016; Chojnowski et al., 2018), b) high throughput screening of candidate proteins (Dittmer et al., 2014) and c) biochemical strategies including lamin A-affinity purification of proteins from either C2C12 myotubes (Depreux et al., 2015), cardiac myocyte (NklTAg) cells or mouse embryonic fibroblasts [Kubben et al., 2010; reviewed by Simon and Wilson (2013)]. Our challenge was therefore two-fold: firstly, to identify lamin A/C proteomes in frailty-relevant native tissues, and secondly, to determine which (if any) associations changed in IL10-KO mice. To test the hypothesis, we immunoprecipitated native lamins A/C and associated proteins from three native tissues in aged (21–22 months old) female mice—heart and skeletal muscle (reported here) and brain (reported separately)—and used Tandem Mass Tags to quantify and compare results from control (C57Bl/6) *versus* IL10-KO mice. After normalizing to the amount of lamin A/C in each sample, most identified proteins were unaffected by IL10-KO, as expected. However, a subset of identified proteins showed differential lamin A/C-association in IL10-KO tissue, relative to controls. Selected candidates-of-interest were displayed as staggered 20-mer synthetic peptides and probed with recombinant lamin A, to screen for direct binding. Results for seven candidates, and molecular mapping of lamin-binding regions, unexpectedly revealed that four new partners (Csrp3, Lmcd1, AldoA, and Perm1) share a proposed lamin-binding motif.

## Materials and methods

### Mouse care and tissue harvest

Female wildtype (WT) C57Bl/6 and IL10-KO (B6.129P2-*IL10* ^*tm1Cgn*^/J) mice were purchased from Jackson Laboratory (Bar Harbor, ME; National Institute on Aging, Bethesda, MD) and housed under specific pathogen-free barrier conditions until the age of 21–22 months in facilities accredited by the Association for Assessment and Accreditation of Laboratory Animal Care International. Nineteen mice (eleven IL10-KO and eight WT) were euthanized by administered inhalation anesthesia in a plastic chamber under a vented hood using gauze soaked in pharmaceutical-grade isoflurane, followed by cervical dislocation once the mouse was unconscious. Death was verified by observed cessation of breathing and heartbeat. Tissues were harvested, weighed, flash-frozen in liquid nitrogen and stored at −80°C as described (Sikka et al., 2013; Malinina et al., 2020). All protocols were approved by the Johns Hopkins University Institutional Animal Care and Use Committee, and all experiments performed accordingly.

### Tissue lysates, immunoprecipitation and Western blotting

Frozen tissues were each pulverized under liquid nitrogen using a pre-chilled mortar and pestle, and powdered tissue was stored at −80C or in liquid nitrogen until use. To prepare lysates, we added powdered tissue (80–100 mg) to 500 uL ice-cold lysis buffer (50 mM Tris-HCl pH 7.4, 300 mM NaCl, 0.3% v/v Triton-X100, 5 mM EDTA, 1 mM DTT, 100 uM PMSF, 1 ug/mL Pepstatin A, 1X Thermo Scientific Halt Protease Inhibitor Cocktail #78430, 50 nM Thiamet G [OGA inhibitor; 1,2-dideoxy-2′-ethylamino-α-d-glucopyranoso-[2,1-*d*]-Δ2′-thiazoline; provided by G. W. Hart) and 50 mM UDP-GlcNAc [Sigma]) in a 2 mL Eppendorf tube on ice, and moved to liquid nitrogen as needed to keep frozen until further processing. Thiamet G and UDP-GlcNAc were included to maintain labile *O*-GlcNAc modifications. Samples were flick-vortexed to mix, incubated 10 min on ice, vortexed 10 s at high speed, then sonicated on ice 20 times (0.5 s bursts) and finally centrifuged 30 min (16,000 *g*, 4°C) to pellet insoluble material. Sonication greatly facilitates solubilization of lamins and associated proteins (Berk et al., 2013; Berk and Wilson, 2016). Supernatant protein concentrations were measured via Bradford assay and adjusted with lysis buffer to 1 ug/uL before use.

#### Immunoprecipitation

For each preparatory immunoprecipitation, 500 uL lysate (500 ug total protein) was incubated with 10 uL anti-lamin A/C mouse mAb 4C11 (Cell Signaling Technologies #4777; 1:50 dilution) with rotation overnight at 4°C. We then added 10 uL Protein G Sepharose slurry (GE Healthcare #17-0618-01, prewashed three times in 300 uL lysis buffer) to each reaction and rotated 1 h at 4°C. After pelleting (1 min, 13,300 *g*, 4°C), the beads were washed three times in 300 uL lysis buffer. For mass spectrometry analysis, bound proteins from each sample were eluted using 50 uL 1% SDS. Alternatively, for SDS-PAGE and Western blotting of smaller scale immunoprecipitations, bound proteins were eluted by heating (95°C) for 5 min in 30 uL of 2x SDS-sample buffer.

#### Analytical SDS-PAGE and Western blots of heart immunoprecipitates

Immunoprecipitates (20 uL each; corresponding to 40 ug input lysate protein) were resolved on Bolt 8% Bis-Tris Plus gels in MOPS running buffer for 5 min at 200 V (20°C–22°C), then at 170 V for 1.5 h (4°C). Resolved proteins were transferred to nitrocellulose membranes for 1.5 h at 300 mA, at 4°C. Membranes were blocked 1 h in blocking buffer (3% BSA, 0.01% Tween-20, 20 mM Tris Base, 137 mM NaCl, pH 7.6). The primary antibody, which specifically recognizes the *O*-GlcNAc modification (IgM mAb CTD110.6, Santa Cruz Biotechnologies, 1:1000; provided by Natasha Zachara) was first diluted into blocking buffer. Blots were then rocked overnight (4°C), washed three times with TBST buffer (0.01% Tween-20, 20 mM Tris Base, 137 mM NaCl, pH 7.6), incubated (1 h at 20°C–22°C) with secondary antibody (HRP-conjugated anti-mouse IgM; Santa Cruz Biotechnology #sc-2064; dilution 1:10,000) in blocking buffer, washed three times in TBST, and finally visualized by enhanced chemiluminescence (Hyblot CL autoradiography film #E3012).

To detect bound lamin proteins, we next incubated with lamin A/C antibodies. Blots were not allowed to dry; each blot was stripped for 10 min in stripping buffer (1.5% w/v glycine, 0.1% w/v SDS, 1% v/v Tween-20, pH 2.2), washed twice (10 min each) in PBS (16 mM Na_2_HPO_4_, 3 mM KH_2_PO_4_, 270 mM NaCl, 5.4 mM KCl, pH 7.4) and twice (5 min each) in TBST. Blots were then blocked 1 h at 20°C–22°C using 3% BSA in TBST, incubated with lamin A/C antibodies (sc-20861 rAb, Santa Crus Biotechnologies, 1:1000 in blocking buffer) overnight at 4°C, then incubated with secondary antibody (HRP-conjugated anti-rabbit IgG; Cell Signaling Technologies #7074S; dilution 1:10,000), washed, and visualized by enhanced chemiluminescence (Hyblot CL autoradiography film #E3012). Films were scanned with the Epson Perfection V500 Photo scanner. Western blot signals were quantified via Quantity One, version 4.6.9.

### Tandem mass tag (TMT) proteomics

Protein extracts (40 ug total at 1 ug/ul in 1% SDS) were reduced with 15 uL of 15 mM DTT for 1 h at 56°C, alkylated by adding 15 uL 100 mM iodoacetamide and incubating in the dark for 30 min, and then TCA/Acetone precipitated. The protein pellet from each sample was digested overnight at 37°C by adding 100 uL Trypsin/LysC mixture [40 ug proteases in 1.2 mL of 100 mM triethylammonium bicarbonate (TEAB; Promega #V5071) or approximately 3.33 ug protease per sample]. Individual samples (40 ug) were labeled with a unique isobaric mass tag reagent (TMT 10-plex, Thermo Scientific) according to manufacturer instructions. Both pairing and labeling order of TMT reagent and peptide sample were randomized. Briefly, the TMT reagents (0.8 ug vials) were allowed to come to room temperature before adding 41 uL anhydrous acetonitrile, then vortexed and centrifuged. The entire TMT reagent vial was added to the 100 ug peptide sample and reacted at room temperature for 1 h. The reaction was quenched by adding hydroxylamine (8 uL) to a final concentration of 5%. All TMT-labeled samples were combined and vacuum centrifuged to dryness removing the entire liquid.

#### Basic reverse phase (bRP) fractionation

Labeled peptide samples were fractionated by basic reverse phase (bRP) chromatography on Oasis HLB uElution plates (Waters). TMT labeled peptides (5%, approximately 20 ug) were bound to HLB resin in 10 mM triethylammonium bicarbonate (TEAB) buffer and step eluted with 0%, 5%, 10%, 25%, and 75% acetonitrile in 10 mM TEAB (0% and 5% fractions were combined). Fractions were dried by vacuum centrifugation.

#### Mass spectrometry analysis

The peptide fractions were resuspended in 20 uL 2% acetonitrile in 0.1% formic acid; approximately 0.5 ug (2 uL) was loaded onto a C18 trap (S-10 uM, 120 Å, 75 um × 2 cm; YMC Co., LTD., Kyoto, Japan) and then separated on an in-house packed PicoFrit column (75 um × 200 mm, 15 um, ±1 um tip, New Objective) with C18 phase (ReproSil-Pur C18-AQ, 3 um, 120 Å, www.dr-maisch.com) using 2%–90% acetonitrile gradient at 300 nL/min over 120 min on a EasyLC nanoLC 1000 (Thermo Scientific). Eluting peptides were sprayed at 2.0 kV directly into an Orbitrap Fusion Lumos (Thermo Scientific) mass spectrometer. Survey scans (full ms) were acquired from 360–1,700 m/z with a cycle time of 3 s. Precursor ions isolated in a 0.7 Da window and fragmented using HCD activation collision energy 39 and 15 s dynamic exclusion, with a scan range of 116 m/z–2,000 m/z. Precursor and fragment ions were analyzed at resolutions 120,000 and 30,000, respectively, with automatic gain control (AGC) target values at 4 × 10^5^ with 50 ms maximum injection time (IT) and 1 × 10^5^ with 118 ms maximum IT, respectively.

#### Data analysis

Isotopically resolved masses in precursor (MS) and fragmentation (MS/MS) spectra were extracted from raw MS data using spectrum selector with recalibration in Proteome Discoverer (PD) software (version 2.4.0.305, Thermo Scientific) and searched using Mascot (2.6.2; www.matrixscience.com) against a *Mus musculus* protein database (RefSeq 2017_83, created 5/23/2019, containing 76,508 sequences. The following criteria were set for all database searches: 1) all species in database; 2) trypsin as the enzyme, 3) two missed cleavages allowed; 4) N-terminal TMT6plex and cysteine carbamidomethylation as fixed modifications; 5) lysine TMT6plex, methionine oxidation, serine, threonine and tyrosine phosphorylation, asparagine and glutamine deamidation, HexNAc on serine or threonine, as variable modifications; and 6) precursor and fragment ion tolerances were set to 5 ppm and 0.03 Da, respectively. Peptide identifications from Mascot searches were filtered at 5% False Discovery Rate (FDR) confidence threshold, based on a concatenated decoy database search, using the Proteome Discoverer. Proteome Discoverer uses only the peptide identifications with the highest Mascot score for the same peptide matched spectrum from the different extraction methods. The protein intensities were reported as S/N of each peptide and relative protein comparisons were calculated using the peptide grouping in Proteome Discoverer. Quan value correction factors were used (Lot TK271715) with a co-isolation threshold of 30. Peptide abundances were normalized against a custom sequence.FASTA file containing only prelamin A [XP_006501136.1 PREDICTED: prelamin-A/C isoform X1 (*Mus musculus*)] to ensure there was no experimental bias in protein quantification that depended on the total amount of lamin A/C immunoprecipitated from each sample.

### Statistical analysis, filtering and curation of proteomic data

Data were analyzed using R version 4.0.4 and all new bioinformatics and statistical analyses described here are available (GitHub repository: https://github.com/aditharun/frailty-laminome). To assess potential differences between the IL10-KO and WT proteomes in heart or muscle, we used a rigorous statistical method developed by Rucinski and others to detect significant changes in protein abundance (Kammers et al., 2015). Starting from the raw peptide spectra data, we removed proteins with an Isolation Interference greater than 30% or which had missing values, yielding Supplementary Table S1. We then normalized the abundances to the lamin A abundance in each channel (i.e., the same sample), and performed a two-sample Student’s t-test using an empirical Bayes method (R script: https://github.com/aditharun/frailty-laminome/blob/main/code/analysis.R). This script produces output spreadsheets that can either include all proteins (Supplementary Table S2), or a subset that excludes as presumed contaminants mitochondrial, ribosomal and keratin proteins. For downstream analysis and succinct reference, we developed a script that matches protein accession numbers with gene names (https://github.com/aditharun/frailty-laminome/blob/main/code/get_gene_names.R) from NCBI (https://www.ncbi.nlm.nih.gov/gene) using the R API for the NCBI Database and searching for the accession number of a particular protein to return a unique identifying number. This number was then matched, also using the NCBI API, to a specific gene name (Supplementary Table S3). Some gene names were unavailable and curated manually in the final table (Supplementary Table S4), which was also curated manually (non-exhaustively) to indicate reported nuclear envelope transmembrane proteins [“NETs”; (Malik et al., 2010)] and nuclear-localized proteins.

The scripts used for volcano plots are available here: https://github.com/aditharun/frailty-laminome/blob/main/code/volcano-stratification.R.

### Peptide array synthesis and probing

Three identical custom peptide arrays were synthesized and printed on cellulose membranes by the Biopolymers and Proteomics Core Facility at the Koch Institute Swanson Biotechnology Center at Massachusetts Institute of Technology (Cambridge MA) as described (Frank and Dübel, 2005), using a Multipep automated peptide synthesizer (INTAVIS Bioanalytical Instruments AG, Koeln, Germany) as described (Frank and Overwin, 1996). The following proteins, all human, were each displayed as synthetic 20-mer peptides with seven amino acid offsets: Fabp4 (132 residues; NP_001433.1), Gins3 (216 residues; NP_073607.2), Perm1 (790 residues; NP_001356826.1), Tmem38a (299 residues; NP_076979.1), AldoA (364 residues; NP_908930.1), Csrp3 (194 residues; P50461-1), Lmcd1 (365 residues; Q9NZU5-1), emerin (254 residues; NP_000108.1), plus other proteins reported separately.

Arrays were incubated with C-terminally His-tagged residues 385–646 of human mature lamin A, affinity-purified from *E. coli* lysates. Protein expression was induced using 1 mM isopropyl-β-D-thio-galactoside (IPTG; 3 h, 37°C), and His-tagged polypeptides were affinity-purified as described (Simon et al., 2010), using Cobalt-charged TALON^®^ metal ion affinity resin (Takara Bio United States, #635502).

Peptide arrays were probed as follows, at room temperature (20°C–22°C) unless otherwise noted. After initial wetting (∼5 min in methanol), each array was washed three times in TBS (pH 7.0) in a polystyrene plate, and then incubated overnight in Membrane Blocking Solution (MBS), made by mixing 20 mL concentrated Casein blocking buffer (Sigma-Genosys #SU-07-250) with 80 mL TBS-T [Tris-Buffered Saline (150 mM NaCl, 20 mM Tris) plus 0.05% (vol/vol) Tween 20; pH 8.0] and 5 g sucrose, then adjusting dropwise with NaOH to final pH 7.0. The arrays were washed twice in TBS-T, then incubated 3 h in 30 mL MBS containing 150 ug recombinant lamin tail protein (final lamin concentration, 190 nM). After two washes in TBS-T, arrays were incubated 1 h in MBS containing 20 uL anti-lamin A/C mouse clone 14 (Millipore Sigma #05-714; 1:1000 dilution), washed twice in TBST and incubated 1 h in MBS containing 6 uL AP-conjugated donkey anti-mouse (Jackson ImmunoResearch #715-055-150; 1-to-5,000 dilution), and finally washed twice in TBS-T. To detect bound antibodies, arrays were washed twice in citrate-buffered saline (CBS: 137 mM NaCl, 2.7 mM KCl, 50 mM citric acid monohydrate, pH 7.0). The arrays were transferred to flat glass trays before adding 20 mL Color Developing Solution, made fresh by adding 80 uL BCIP solution [made by dissolving 60 mg BCIP (5-bromo-4-chloro-3-indolylphosphate p-toluidine salt; Sigma #51K1567) per mL absolute dimethylformamide (DMF; Cell Signaling Technologies #12767)] to 120 uL MTT solution [made by dissolving 50 mg of 3-(4,5-Dimethylthiazol-2-yl)-2,5-Diphenyltetrazolium Bromide (MTT) per mL 70% v/v DMF in water] and 100 uL MgCl2 (1 M stock) to 20 mL CBS. Note this specific form of BCIP is crucial to remove the purple color for re-use. The array was then incubated 30–35 min in Color Developing Solution, watching for color development, washed twice in PBS to stop the reaction, and imaged using an Azure Biosystems C600 imager, with the RGB capture setting (Cy2, Cy3, Cy5) and autoexposure. Arrays were stored at 4°C in PBS until the next stage, when they were stripped to remove lamins, antibodies and purple color in a multi-step process: 1) washed twice in 20 mL ddH2O, 2) incubated in 20 mL DMF until the purple color disappeared (typically ∼10 min), 3) washed three times in ddH_2_O, 4) transferred into a plastic “pouch” (Kapak by Ampac, 404-24) and washed three times (10 min each) in ∼30 mL Stripping Mix A (PBS, pH 7.0, containing 8 M Urea, 1% SDS and 0.5% beta-mercaptoethanol) in a 40°C sonication bath, 5) washed three times in Stripping Mix B (10% acetic acid, 50% ethanol, in ddH_2_O), and 6) washed three times in 100% ethanol. The stripped array was then imaged and stored at 4°C in PBS until the next cycle of probing and stripping.

## Results

The hearts, hind limb skeletal muscle and brains from a total of 19 age-matched (21–22 months old) female c57Bl/6 controls (8 mice) and IL10-KO (11 mice) were harvested, weighed and flash-frozen in liquid nitrogen until use. We prepared whole-tissue protein lysates, sonicating to improve solubilization of lamin networks, and used mouse mAb 4C11, raised against Ig-fold residues 400–550 (shared by lamin A and lamin C) to coimmunoprecipitate native lamins A/C and associated proteins. To determine which ten mice to select for quantitative multiplex mass spectrometry analysis, we used SDS-PAGE to resolve small aliquots of heart immunoprecipitates from all IL10-KO mice (samples 1–11) and all WT control mice (samples 12–19; Supplementary Figure S1). We immunoblotted first with antibody CTD_110.6_, specific for *O*-GlcNAc modifications on Ser/Thr, to query potential hyper-*O*-GlcNAcylation of lamin A (Supplementary Figure S1, α-*O*-GlcNAc), then stripped and re-probed with antibody SC20681 to detect lamin A and lamin C (Supplementary Figure S1, α-lamin A/C). Both lamins were detected in heart lysates, as expected (Afilalo et al., 2007; Cattin et al., 2015), with similar total (A plus C) signals in IL10-KO and WT hearts. Although the *O*-GlcNAc-to-lamin A signals trended higher in IL10-KO hearts (Supplementary Figure S1), this was inconclusive because we could not rule out *O*-GlcNAcylation of proteins that might have co-migrated with lamin A in SDS-PAGE.

We then selected the five mice of each genotype (IL10-KO, control) whose heart, muscle and brain samples moved forward to mass spectrometry analysis, mainly ruling out mice with tumors (mice #2, 5, 11) or skewed/atypical *O*-GlcNAc signals (mice #1, 7, 15, 18; Supplementary Figure S1). Heart and skeletal muscle proteomes are reported here. Brain yielded the largest proteome (>2,400 candidates) and will be reported separately. In addition, intrigued by the exceptionally high *O*-GlcNAc signal in the heart from IL10-KO mouse #005 (Supplementary Figure S1), we identified its lamin A/C heart proteome separately, as a case study, without quantification. Mouse #005 had an adrenal tumor, and its heart proteome included many proteins not found in the other ten hearts (data not shown). One such protein, Phf2 (PHD finger protein 2), is a demethylase that derepresses inflammatory genes (Baba et al., 2011; Stender et al., 2012; Park et al., 2016), and can be recruited to promotors by NFκB (p65) to repress transcription (Shi et al., 2014). Because Phf2 was detectably lamin-associated only in a heart that was both chronically inflamed and chronically stressed, presumably due to tumor-derived adrenaline “fight or flight” signaling, we speculate that adrenal signaling alone or in combination with inflammation might promote Phf2 association with A-type lamins, and thereby contribute to de-repression of inflammatory genes. We wonder if Phf2 might be relevant to a patient with adrenal Cushing syndrome who experienced multiple *LMNA* (p.R545H)-associated laminopathies (Guillín-Amarelle et al., 2018), or during idiopathic inflammatory myopathy (Komaki et al., 2011; Moraitis et al., 2015) or other stress conditions.

### Identification and quantification of native lamin A/C proteomes in heart and skeletal muscle

Proteins immunoprecipitated from each sample were uniquely covalently marked using Tandem Mass Tags (TMT; Thompson et al., 2003), a stable isotope-based approach that allows all ten samples from the same tissue (e.g., hearts) to be pooled for multiplex analysis and quantification by mass spectrometry. Potential changes in lamin A/C association were measured after normalizing each protein to the lamin A/C signals in the same sample. Significant log2-fold changes in protein abundance were quantified based on normalized WT-to-IL10-KO signal ratios using the empirical Bayes method described by Rucinski and others (Kammers et al., 2015). Raw results for all identified proteins in skeletal muscle and heart are provided in Supplementary Table S1, and normalized results in Supplementary Table S2.

To focus further analysis, proteome datasets were filtered by removing ribosomal proteins, keratins and most mitochondrial proteins as presumed contaminants (Supplementary Table S3). Sarcomeric proteins, which are highly abundant and relatively insoluble in heart and muscle, were also presumed contaminants of immunoprecipitation but were not filtered out. Our final filtered Supplementary Table S4 was curated manually to note proteins that were: 1) known or predicted (Cytoscape version 3.8.2) to localize in the nucleus, 2) reported to bind lamin A/C directly, or 3) identified as nuclear envelope integral membrane proteins (“NETs”). NET classification was based on biochemical isolation of native NETs from liver, white blood cells or C2C12 myoblasts [Supplementary Table S4 in Korfali et al. (2010), Korfali et al. (2012), Worman and Schirmer (2015)], or from cultured mesenchymal stem cells, adipocytes or myocytes [Supplementary Tables S1, S2 in Cheng et al. (2019)].

### Overview of native lamin A/C proteomes: 62 proteins identified in both heart and skeletal muscle

**FIGURE 1:**
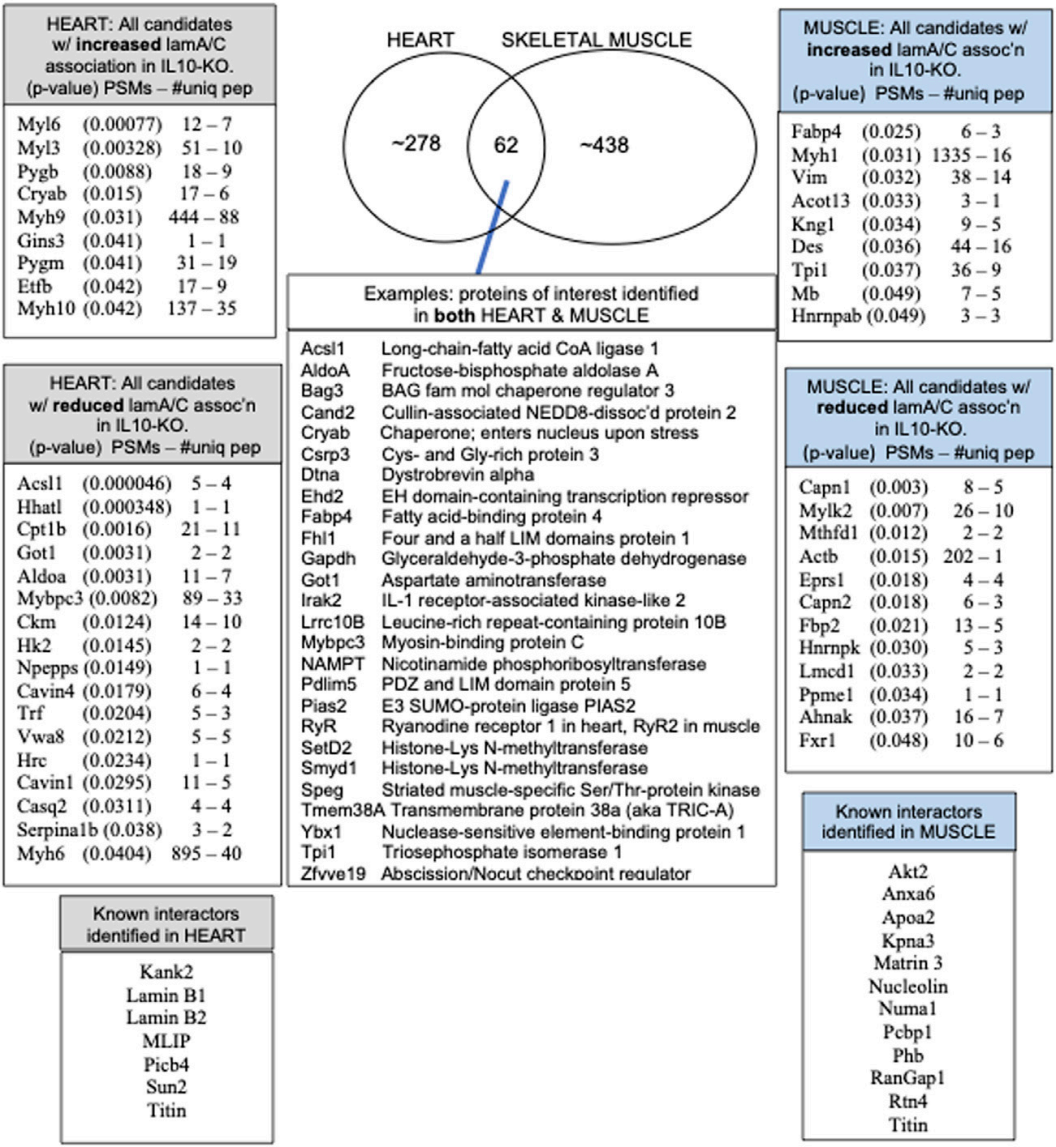
Overview and highlights from the heart and muscle proteomes, including the number of peptide spectra (PSMs) and number of unique peptides (PSMs—#uniq) used to identify and quantify each protein that differed significantly in heart or muscle after normalization to lamins A/C in all ten IL10-KO samples.

We identified ∼340 proteins in heart and ∼500 proteins in skeletal muscle with high confidence (Supplementary Table S4). Sixty-two proteins were identified in both heart and muscle (examples shown in Figure 1), including chromatin regulators Smyd1 [adds the repressive H3K4me “mark” on histone H3 (Tracy et al., 2018)] and SetD2 [adds the H3K36me3 mark; (McDaniel and Strahl, 2017)], dystrobrevin-alpha [Dtna; (Aguilar et al., 2015)]; and Tmem38a (aka “Trimeric intracellular cation channel type A”), a nuclear membrane protein required for muscle-specific 3D genome organization (Robson et al., 2016) and genetically linked to Emery-Dreifuss muscular dystrophy (Meinke et al., 2020). Additional proteins identified in both tissues included NAMPT [controls the rate-limiting step in NAD biosynthesis, and the release of Clock-Arntl/BMAL1 heterodimers from Sirt1-mediated repression; (Ramsey et al., 2009); (Maynard et al., 2022)], Gapdh [glyceraldehyde-3-phosphate dehydrogenase; enters the nucleus during glucose starvation (Chang et al., 2015) and autophagy (Iqbal et al., 2021)], transcription factor Fhl1 (Roux et al., 2012) which interacts with emerin and lamin A and is genetically linked to EDMD (Ziat et al., 2016), and shuttling transcription factor Csrp3 [“muscle LIM protein”; a MyoD1 co-factor that enters the nucleus in response to mechanical force, and protects against muscular dystrophy (Mathiesen et al., 2019)].

A small number of nuclear envelope integral membrane proteins (“NETs”) and characterized lamin A/C-binding proteins were identified in the heart, including MLIP (Ahmady et al., 2011), Plcb4 (Dittmer et al., 2014), transcription regulator Kank2 (Dittmer et al., 2014), Sun2 [NET; (Haque et al., 2010)] and RyR2 [ryanodine receptor 2; (Kapiloff et al., 2001; Dridi et al., 2021)]. In skeletal muscle we identified Ryr1 [Ryanodine receptor 1; (Dridi et al., 2021), Matrin 3 (Depreux et al., 2015) and lamin A-proximal proteins Numa1, Pcbp1 (poly(rC)-binding protein, aka HnrnpE1)] and RanGap1 (Roux et al., 2012; Supplementary Table S4). Other expected partners such as emerin were not recovered from striated muscle, but were identified in brain, a soft tissue.

### Most identified proteins were not significantly affected in the IL10-KO model of frailty

**FIGURE 2:**
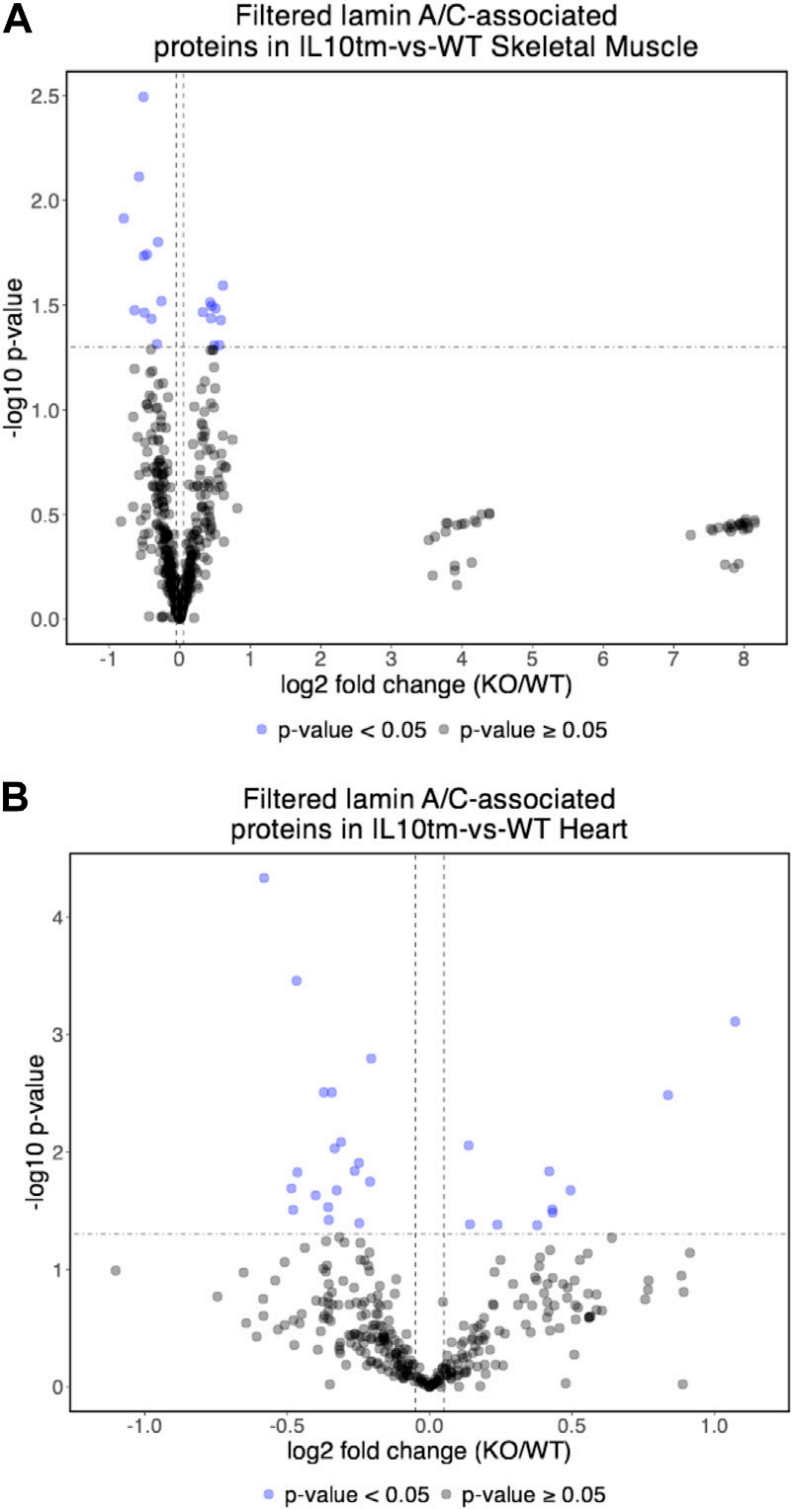
**(A,B)** Volcano plot of lamin A/C-associated proteins in mouse skeletal muscle **(A)** and mouse hearts **(B)**. In both tissues, relatively few identified proteins were affected significantly (<0.05) in the IL10-KO (here denoted as “IL10^tm^”) tissue.

Most proteins identified in this study were not detectably affected in IL10-KO tissues, as seen in volcano plots for skeletal muscle (Figure 2A) and heart (Figure 2B). This was encouraging because it suggested these proteins reproducibly co-precipitated with native A-type lamins, whether due to genuine association (direct or indirect) or artifact. Curated examples of IL10-KO-unaffected proteins are shown for skeletal muscle in Table 1, and for heart in Table 2. Proteins unaffected by chronic inflammation (IL10-KO) were still of great interest as candidate (novel) partners for A-type lamins in these native tissues. Skeletal muscle candidates included Rragd [regulates mTORC1; (Tsun et al., 2013)], condensin subunits NCAPH (condensin complex subunit 2) and Smc3 (structural maintenance of chromosomes protein 3), chromatin repressor Sirt2 (NAD-dependent protein deacetylase sirtuin-2), scaffolding proteins 14-3-3 epsilon (Ywhae) and 14-3-3 gamma (Ywhag), and transcription factors Bin1 (Myc box-dependent-interacting protein 1), smoothelin, Thrap3 (thyroid hormone receptor-associated protein 3), Dmrt (doublesex- and mab-3-related transcription factor 2), Stat5B and Fhl3 (Table 1), and signaling kinase Akt2 (which can phosphorylate lamin A; Fan et al., 2023). Others included MAP “kinase kinases” (Map2k3, Map2k4, and Map2k6), multiple subunits of the 5′-AMP-activated protein kinase complex (Prkaa2, Prkab2, Prkag1, Prkaca, and Prkar2a), Ca++/calmodulin-dependent protein kinase type II (Camk2a and Camk2g), the protein phosphatase 1 complex (Ppp1cc, Ppp1r12b, Ppp1r3a), as well as protein phosphatase 1B (Ppm1b), protein phosphatase 2 (Ppp2cb), and “TRiC” chaperonin complexes [T-complex protein 1, Cct2/beta, Cct3/gamma, Cct4/delta, Cct7/eta, Cct8/theta; (Jin et al., 2019); Supplementary Table S4]. TRiC complexes are huge (∼1,000 kD) and might have co-precipitated as an artifact of density, but also reportedly associate with heterochromatin (Souès et al., 2003) and function in the nucleus (Pejanovic et al., 2012). Other novel candidates from heart included transcription factor Ndrg2, which protects against ischemia-reperfusion injury (Sun et al., 2013), several kinases (e.g., Speg, Skp1, Taok1, Sgk223), Pias2 (E3 SUMO-protein ligase), Mad1l1 (mitotic spindle checkpoint protein MAD1) and Ppp2ca (Ser/Thr-protein phosphatase 2A catalytic subunit alpha), which regulates an essential lamin-binding protein named Barrier to Autointegration Factor 1 (BANF1) and influences postmitotic nuclear assembly (Ahn et al., 2019).

**TABLE 1:** Selected skeletal muscle candidates unaffected by IL10-KO.

Gene	Protein name
Ugp2	UTP--glucose-1-phosphate uridylyltransferase
Vdac3	Voltage-dependent anion-selective channel protein 3
Cavin2	Caveolae-associated protein 2
Apobec2	C- > U-editing enzyme APOBEC-2
Ncaph	Condensin complex subunit 2
Faf1	FAS-associated factor 1
Dnajb4	DnaJ homolog subfamily B member 4
Zbed5	SCAN domain containing 3
Dmrt2	Doublesex- and mab-3-related transcription factor 2
Irak2	Interleukin-1 receptor-associated kinase-like 2 isoform a
Tex55	Uncharacterized protein C3orf30 homolog
Stat5b	Signal transducer and activator of transcription 5B
Alpk3	Alpha-protein kinase 3
Mettl14	N6-adenosine-methyltransferase subunit METTL14
Anp32a	Acidic leu-rich nuclear phosphop’n 32 family member A
Krt90	Uncharacterized protein LOC239673
Fhl3	Four and a half LIM domains protein 3
Ube2m	NEDD8-conjugating enzyme Ubc12 isoform 1
Sh3d21	SH3 domain-containing protein 21
Ube2n	Ubiquitin-conjugating enzyme E2 N
Park7	Protein DJ-1
Thrap3	Thyroid hormone receptor-associated protein 3
Sirt2	NAD-dependent protein deacetylase sirtuin-2
Ankrd2	Ankyrin repeat domain-containing protein 2
Prdm2	PR domain zinc finger protein 2
HnrnpU	Heterogeneous nuclear ribonucleoprotein U
Tardbp	TAR DNA-binding protein 43
Pdlim7	PDZ and LIM domain protein 7
Klhl41	Kelch-like protein 41
Prag1	Tyrosine-protein kinase SgK223
Fkbp1a	Peptidyl-prolyl cis-trans isomerase FKBP1A
Rragd	Ras-related GTP-binding protein D
Hdgf	Hepatoma-derived growth factor
Mybpc1	Myosin-binding protein C, slow-type
Marcks	Myristoylated alanine-rich C-kinase substrate
Osbp	Oxysterol-binding protein 1
Pdlim5	PDZ and LIM domain protein 5 isoform ENH2
Bin1	Myc box-dependent-interacting protein 1
Ywhag	14-3-3 protein gamma
Smtn	Smoothelin
Fam114a2	Protein FAM114A2
Myo18b	Unconventional myosin-XVIIIb
Tacc2	Transforming acidic coiled-coil-containing protein 2
Mindy1	De-ubiquitinating enzyme

**TABLE 2:** Selected heart candidates unaffected by IL10-KO.

Gene	Protein name
Eef2	Elongation factor 2
Hsp90ab1	Heat shock protein HSP 90-alpha beta 1
HNRNPC	Heterogeneous nuclear ribonucleoproteins C (C1/C2)
Speg	Striated muscle-specific serine/threonine-protein kinase
Bag3	BAG family molecular chaperone regulator 3
Irak2	Interleukin-1 receptor-associated kinase-like 2
Chchd3	MICOS complex subunit Mic19
HNRNPM	Heterogeneous nuclear ribonucleoprotein M
HNRNP2b1	Heterogeneous nuclear ribonucleoproteins A2/B1
Fabp4	Fatty acid-binding protein, adipocyte
Ryr2	Ryanodine receptor 2
Taf15	TATA-binding protein-associated factor 2N
Skp1	S-phase kinase-associated protein 1
Sorbs2	Sorbin and SH3 domain-containing protein 2
Ndrg2	Protein NDRG2 (N-myc downstream-regulated gene 2)
IGTP	Interferon gamma induced GTPase
Ndufa13	NADH dehydrogenase
Phf5a	PHD finger-like domain-containing protein 5A
ETV3L	ETS translocation variant 3-like protein
Perm1	PGC-1 and ERR-induced regulator in muscle protein 1
Ybx1	Nuclease-sensitive element-binding protein 1
Pdlim5	PDZ and LIM domain protein 5
Fam210a	Protein FAM210A
Ppp2ca	Serine/threonine-protein phosphatase 2A catalytic subunit
Fhl2	Four and a half LIM domains protein 2
Prdm2	PR domain zinc finger protein 2
Bcl9	B cell CLL/lymphoma 9 protein
Sorbs1	Sorbin and SH3 domain-containing protein 1
Lrrfip2	Leucine-rich repeat flightless-interacting protein 2
Tmem38a	Trimeric intracellular cation channel type A
Smyd1	Histone-lysine N-methyltransferase Smyd1
Msn	Moesin
Gapdh	Glyceraldehyde-3-phosphate dehydrogenase
Ndrg2	protein NDRG2
Csrp3	Cysteine and glycine-rich protein 3 (aka MLP)
Ehd2	EH domain-containing protein 2
Pias2	E3 SUMO-protein ligase PIAS2
Lmo7	LIM domain only protein 7
Sh3d21	SH3 domain-containing protein 21
Setd2	Histone-lysine N-methyltransferase SETD2
Kiaa2018/USF3	Basic helix-loop-helix domain-containing protein USF3
Taok1	Ser/Thr-protein kinase TAO1
Slc25a5	ADP/ATP translocase 2
Cand2	Cullin-associated NEDD8-dissociated protein 2
Sgk223	Tyrosine-protein kinase SgK223
Mad1l1	Mitotic spindle assembly checkpoint protein MAD1

### Two unaffected heart candidates, Perm1 and Fam210A, are relevant to frailty phenotypes

Two proteins, Perm1 (PGC-1 and ERR-induced regulator in muscle protein 1) and Fam210A, were unaffected in IL10-KO hearts but intrinsically interesting as frailty-relevant candidates. Perm1 is an intrinsically disordered protein, highly expressed in heart and skeletal muscle, that regulates genes required for endurance exercise, mitochondrial biogenesis and oxidative capacity in muscle (Cho et al., 2016; Cho et al., 2019; Cho et al., 2021). Perm1 localizes primarily at sites of mitochondrial-ER contact but also enters the nucleus and promotes transcription of genes required for fatty acid oxidation (Huang et al., 2022). Perm1(aka C1orf170) showed selective binding to “progerin,” the toxic internally deleted form of prelamin A, in a yeast two-hybrid study (Dittmer et al., 2014). The other frailty-relevant candidate, Fam210A is genetically linked to human sarcopenia, bone fractures and reduced grip strength (Tanaka et al., 2018; Trajanoska et al., 2018; Tanaka et al., 2020). Fam210A regulates mitochondrial-encoded gene expression and localizes both in mitochondria and the cytoplasm but is relatively uncharacterized as a protein (Wu et al., 2021).

### A subset of candidates had significant changes in lamin A/C association in IL10-KO tissue

Certain candidates identified in both tissues were affected by IL10-KO in one tissue. For example, metabolic regulators Fabp4 and Tpi1, with opposite effects on the “browning” of white fat [Fabp4 inhibits; Tpi1 promotes; (Liu et al., 2022)], were both significantly increased in IL10-KO muscle (*p* < 0.0255 for Fabp4; *p* < 0.0374 for Tpi1; Table 1). Tpi1 controls nuclear acetate levels and influences global histone acetylation (Zhang et al., 2021). Cryab (aka Hspb5), which enters the cardiomyocyte nucleus in response to non-damaging endurance exercise (Antonioni et al., 2020), was identified in both tissues and showed higher association in IL10^tm^ hearts (*p* < 0.0146; Table 2). AldoA (Aldolase 1A retrogene 1), which has central roles in glycolysis and gluconeogenesis in the cytoplasm and regulates ribosome biogenesis in the nucleus (Schwarz et al., 2022), showed reduced association in IL10-KO hearts (*p* < 0.0031; Supplementary Table S4).

### ∼21 candidates identified only in skeletal muscle were affected by IL10-KO

The subset of proteins from skeletal muscle that changed significantly (*p* < 0.05) in the frailty model are all listed in Figure 1 and detailed in Supplementary Table S4. Lamin A/C association was significantly reduced for Lmcd1 (*p* < 0.033), a transcription factor that increases skeletal muscle mass (Ferreira et al., 2019)*,* and three other proteins of interest: Ppme1 [*p* < 0.034; methylates and inhibits protein phosphatase 2A (Xing et al., 2008; Kauko et al., 2020) and colocalizes with lamin A/C (Pokharel et al., 2015)]; Fxr1 (Fragile X syndrome-related protein 1; *p* < 0.049, Table 1), and Fbp2 (fructose-1,6-bisphosphatase isozyme 2; *p* < 0.021), a mitochondrial protein that also binds c-Myc and represses c-Myc-dependent transcription of TFAM, a master (positive) regulator of mitochondrial gene expression, in the nucleus (Huangyang et al., 2020).

### ∼30 candidates identified only in heart were affected by IL10-KO

The subset of proteins from heart that changed significantly (*p* < 0.05) in the frailty model are all listed in Figure 1 and detailed in Supplementary Table S4 *.* Lamin A/C association in heart was reduced for ER membrane protein HHATL (protein-cysteine N-palmitoyltransferase HHAT-like protein; *p* < 0.00034) and two mitochondrial proteins: Acsl1 (long-chain-fatty-acid CoA ligase 1; *p* < 0.00005) and Cpt1B (carnitine O-palmitoyltransferase 1; *p* < 0.0016; Table 2; see Discussion). Lamin A/C association increased for Myl6 (myosin light chain 6; *p* < 0.00076), Pygb (glycogen phosphorylase, brain isoform; *p* < 0.0088; Uno et al., 1998; enters nucleus: Sun et al., 2019), Cryab (*p* < 0.015), Aldoa (*p* < .0.003) and Gins3 (3.5-fold increase; *p* < 0.041; Table 2). Gins3 is a subunit of DNA helicase complexes (Kamada, 2012) that also regulates myocardial repolarization (Milan et al., 2009; Newton-Cheh et al., 2009) and is downregulated in metabolically unhealthy obese adults (Das et al., 2015).

### Rationale and overview of peptide array screening strategy

There were many reasons why protein association with lamin A/C might have changed in IL10-KO tissues, from altered mRNA expression to changes in the posttranslational modifications, nuclear localization or stability of any given candidate protein. We therefore chose a proof-of-principal validation question: did these native proteomes include any novel lamin A-binding proteins? We knew it was feasible to use recombinant lamin A “tails” (residues 385–646) as probes to detect binding to SDS-PAGE-resolved partners such as emerin (Lee et al., 2001), and we previously used peptide arrays to map sites of emerin-emerin interaction (Berk et al., 2014) that were validated in mechanically stressed cells (Fernandez et al., 2022). We therefore chose a peptide array strategy to test candidate binding to recombinant lamin A.

**FIGURE 3:**
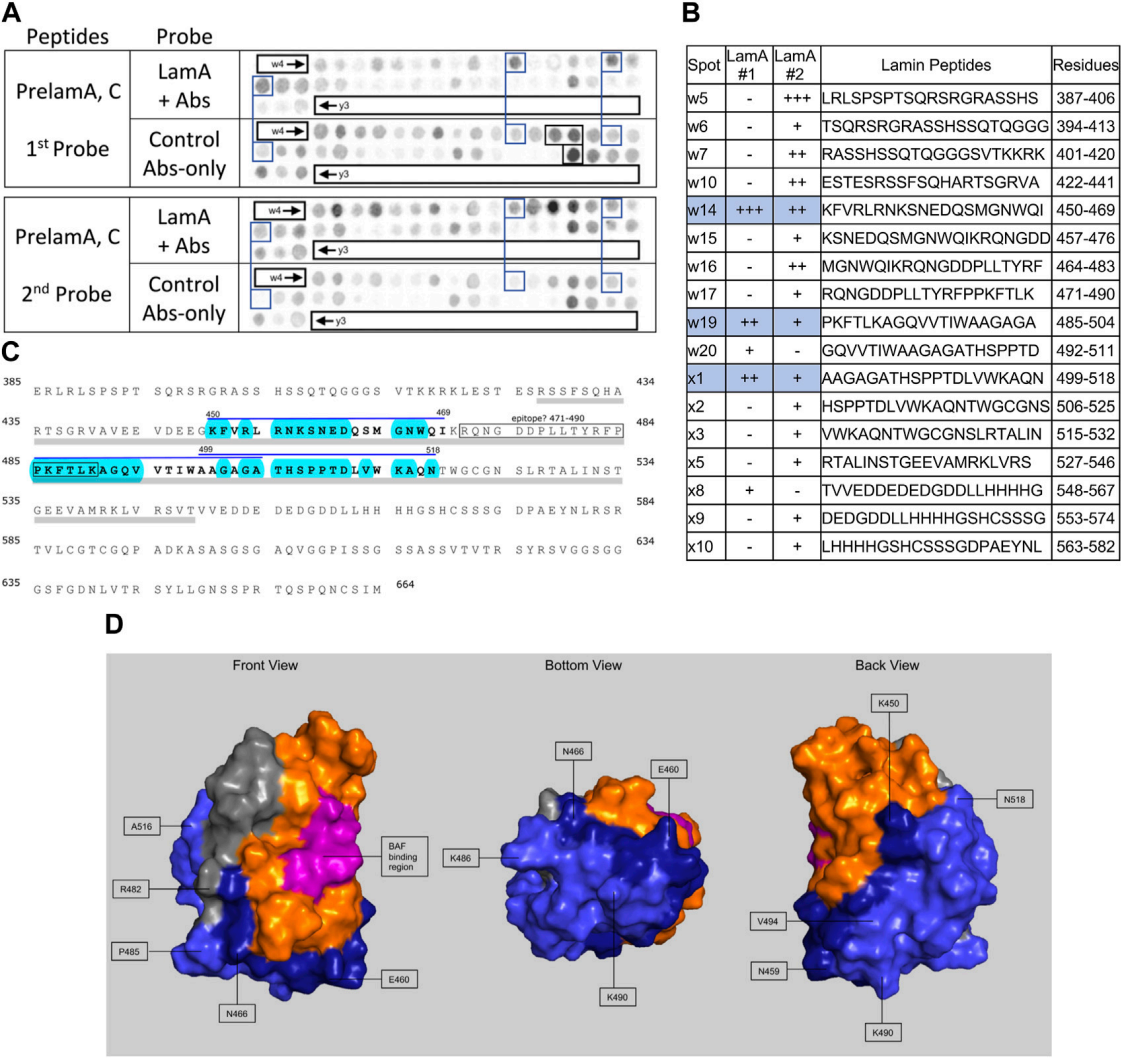
Recombinant lamin A binding to arrayed prelamin A peptides. **(A)** Peptide array results from two independent experiments. “first probe” shows two identical arrays, one probed with lamin A protein and detected using primary (lamin A/C) and secondary antibodies, the other (“control”) probed only with detecting antibodies. Paired blue boxes indicate convincing positive spots and corresponding controls. Black boxes indicate antibody-only signals, considered as possible epitopes for the detecting primary anti-lamin A/C antibody. **(B)** Table summarizing peptide array results, listing each prelaminA peptide to which lamin A bound weakly (+), moderately (++) or strongly (+++) above background in each experiment, and their amino acid sequence and positions in the full-length protein. Convincing positives are shaded blue in the table and indicated by a blue bar in the amino acid sequence. **(C)** Amino acid sequence of human prelamin A residues 385–664. The gray bar indicates Ig-fold residues 428–549. Residues from convincing positives are bold, with a blue overline. Blue shading indicates residues that are solvent-exposed in the atomic structure. **(D)** Pymol surface views of the Ig-fold domain (PDB accession number 1IVT). Residues in lamin-binding peptides are shaded blue; residues that are visible in these surface views are therefore solvent-exposed and hypothetically accessible in the context of the full protein. Dark blue indicates residues from peptide w14 (strongest signals). Light blue indicates residues from peptides w19 and x1. Magenta, BAF-interacting residues (Samson et al., 2018). Gray indicates deduced location of the epitope recognized by the antibody used to detect bound lamins on peptide arrays.

**FIGURE 4:**
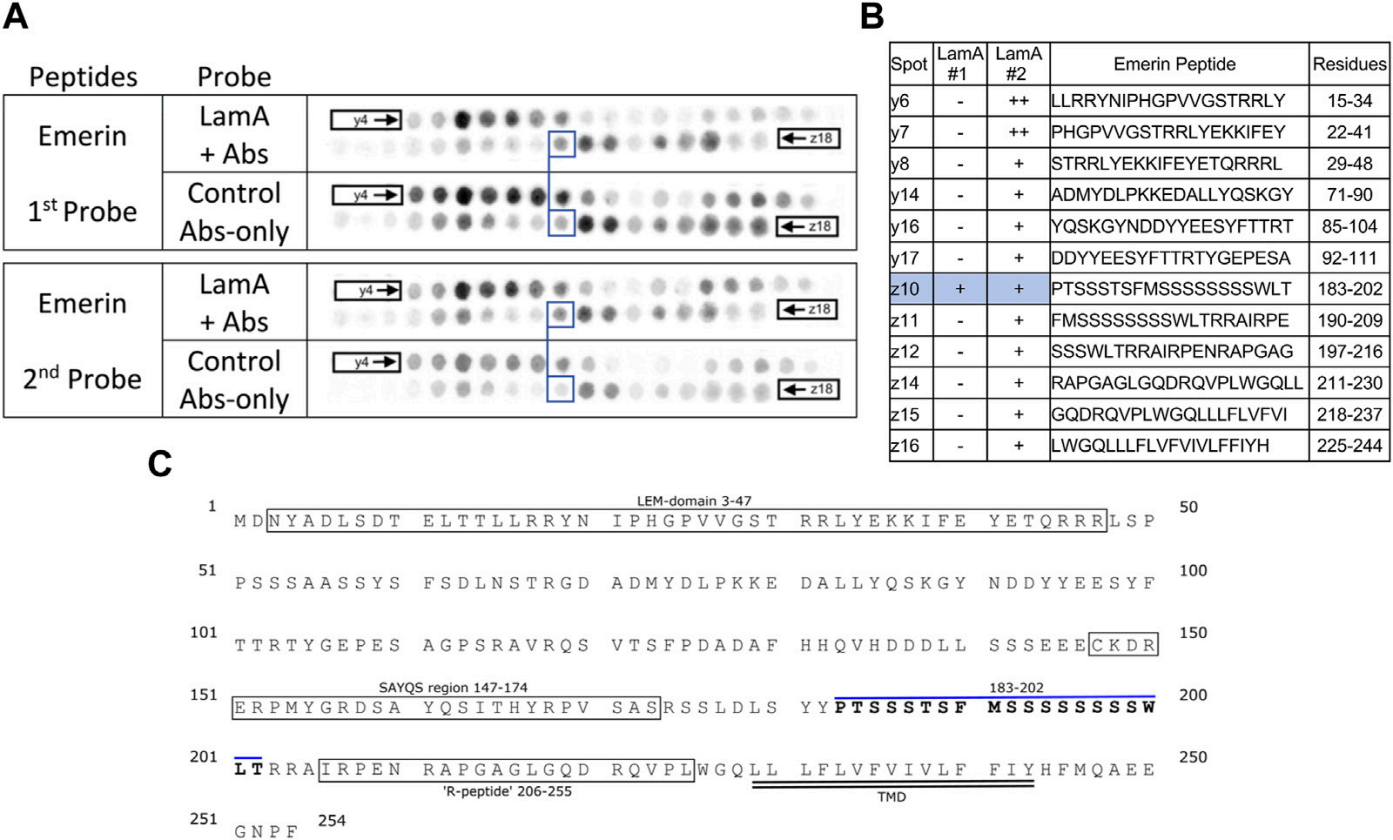
Recombinant lamin A binding to arrayed emerin peptides. **(A)** Peptide array results from two independent experiments. “first probe” shows two identical arrays, one probed with lamin A protein and detected using primary (lamin A/C) and secondary antibodies, the other (“control”) probed only with detecting antibodies. Paired blue boxes indicate convincing positive spots and corresponding controls. **(B)** Table summarizing peptide array results, listing each emerin peptide to which lamin A bound weakly (+), moderately (++) or strongly (+++) above background in each experiment, and their amino acid sequence and positions in the full-length protein. Convincing positives are shaded blue in the table and indicated by a blue bar in the amino acid sequence. **(C)** Amino acid sequence of human emerin residues 1–254. Boxes indicate the LEM-domain fold and previously characterized functional regions, “SAYQS region” and “R-peptide”, that mediate emerin homo-oligomerization [see schematics in Berk et al. (2014)]. Double underlines indicate the transmembrane domain (TMD). Residues from convincing positives are bold, with a blue overline. We did not use blue shading, or show an atomic structure of the LEM-domain, because emerin is intrinsically disordered outside the LEM-domain and the identified lamin-binding peptide is in the disordered region.

**FIGURE 5:**
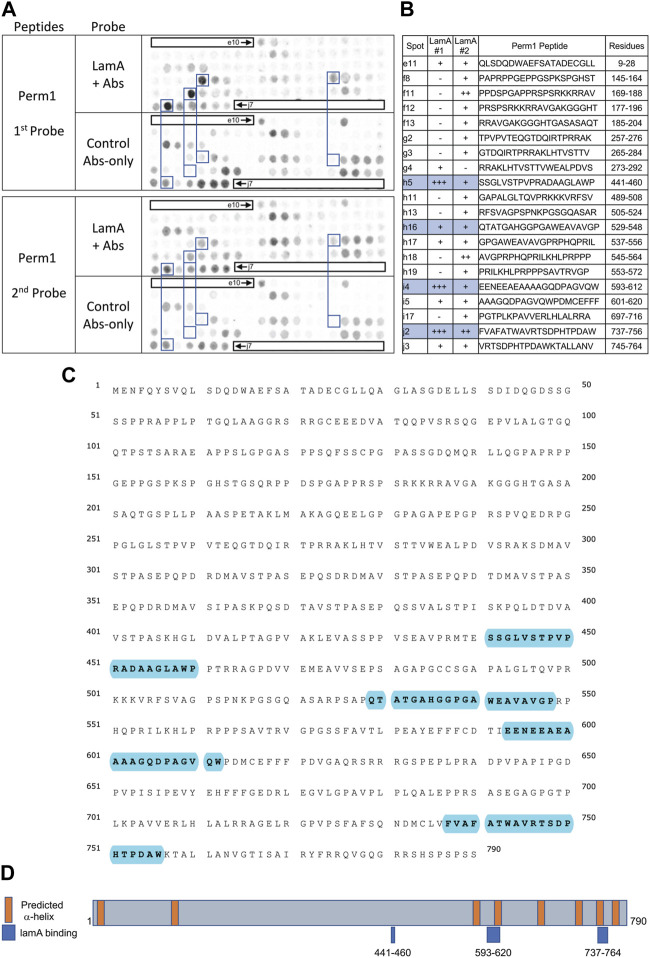
Recombinant lamin A binding to arrayed Perm1 peptides. **(A)** Peptide array results from two independent experiments. “first probe” shows two identical arrays, one probed with lamin A protein and detected using primary (lamin A/C) and secondary antibodies, the other (“control”) probed only with detecting antibodies. Paired blue boxes indicate convincing positive spots and corresponding controls. **(B)** Table summarizing peptide array results, listing each human Perm1 peptide to which lamin A bound weakly (+), moderately (++) or strongly (+++) above background in each experiment, and their amino acid sequence and position in the full-length human Perm1 protein. **(C)** Amino acid sequence of human Perm1; blue shading indicates lamin-binding peptides. **(D)** Schematic depicting human Perm1 residues 1–790. Orange indicates predicted α-helices; blue indicates lamin-binding peptides. No structure is shown because Perm1 is intrinsically disordered.

**FIGURE 6:**
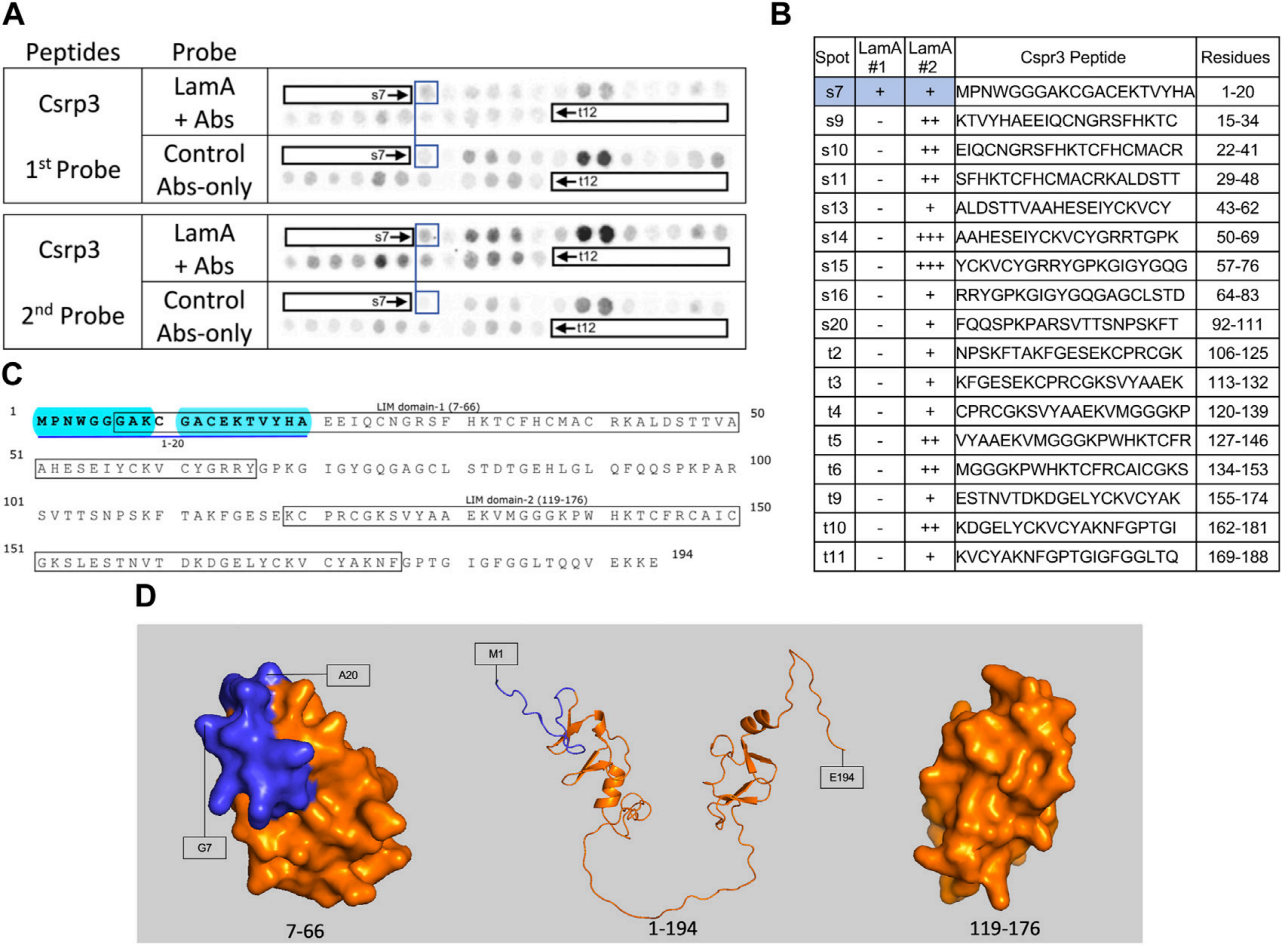
Recombinant lamin A binding to arrayed Csrp3 peptides. **(A)** Peptide array results from two independent experiments. “first probe” shows two identical arrays, one probed with lamin A protein and detected using primary (lamin A/C) and secondary antibodies, the other (“control”) probed only with detecting antibodies. Paired blue boxes indicate convincing positive spots and corresponding controls. **(B)** Table summarizing peptide array results, listing each Csrp3 peptide to which lamin A bound weakly (+), moderately (++) or strongly (+++) above background in each experiment, and their amino acid sequence and position in the full-length protein. Convincing positives are shaded blue in the table and indicated by a blue bar in the amino acid sequence. **(C)** Amino acid sequence of human Csrp3 residues 1–194. Boxes indicate the two LIM-domains. Residues in the one convincing positive are bold, with a blue underline. Blue shading indicates residues that are disordered (presumed exposed), or solvent-exposed in the NMR structure. **(D)** NMR surface views of LIM domain-1 (PDB 2010-NMR; residues 7–66) and LIM-domain-2 (PDB 2013-NMR; residues 119–176) in Csrp3 are shown on the left and right panels, respectively. Middle panel shows the Alphafold-predicted ribbon structure of Csrp3 including disordered central and terminal regions. Dark blue indicates residues in lamin-binding peptide s7, most of which are visible here, hence solvent-exposed and hypothetically accessible in the full protein.

**FIGURE 7:**
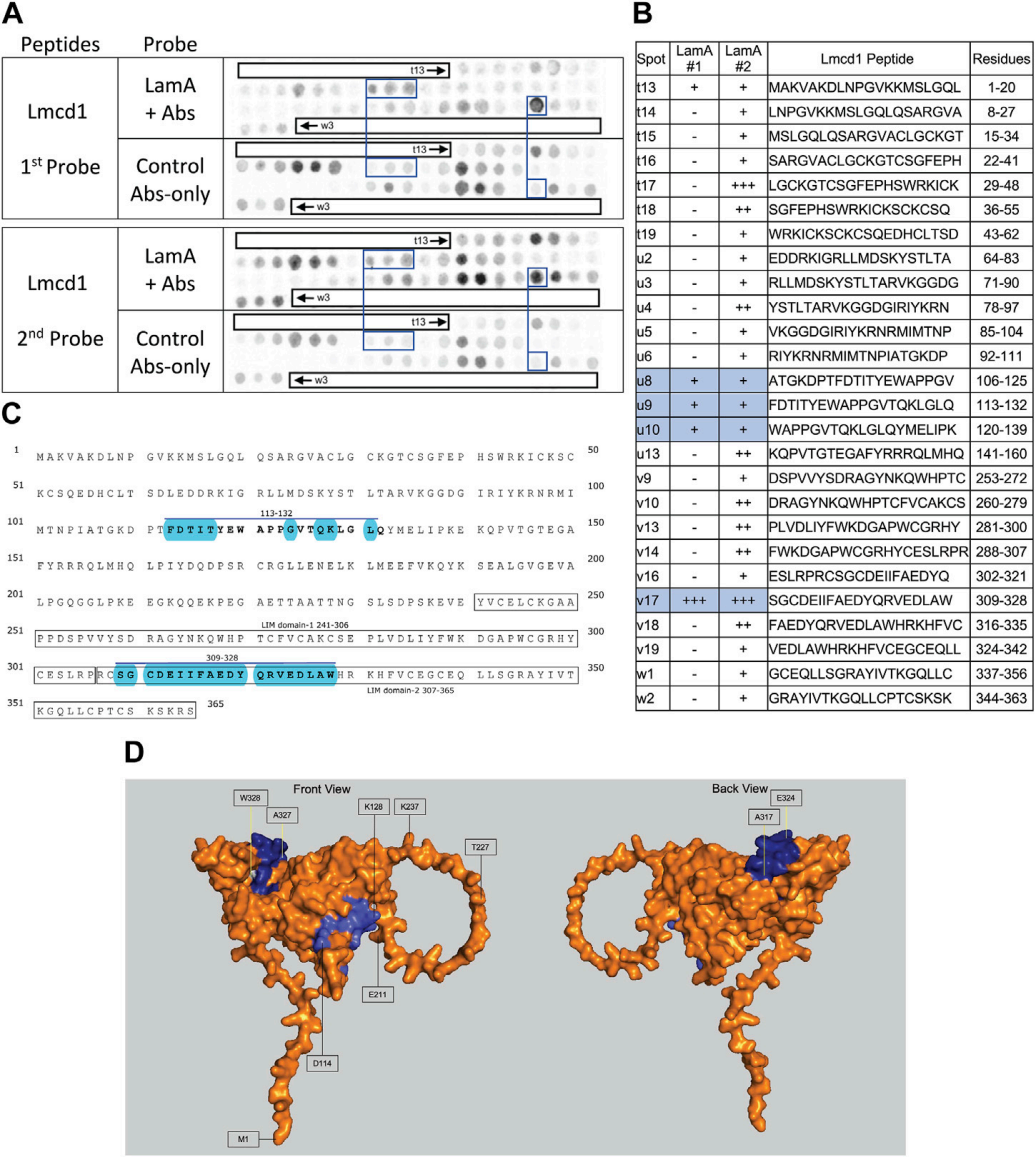
Recombinant lamin A binding to arrayed Lmcd1 peptides. **(A)** Peptide array results from two independent experiments. “first probe” shows two identical arrays, one probed with lamin A protein and detected using primary (lamin A/C) and secondary antibodies, the other (“control”) probed only with detecting antibodies. Paired blue boxes indicate convincing positive spots and corresponding controls. **(B)** Table summarizing peptide array results, listing each Lmcd1 peptide to which lamin A bound weakly (+), moderately (++) or strongly (+++) above background in each experiment, and their amino acid sequence and position in the full-length protein. Convincing positives are shaded blue in the table and indicated by a blue bar in the amino acid sequence. **(C)** Amino acid sequence of human Lmcd1 residues 1–365. LIM domain-1 and LIM domain-2 are boxed. Residues in the two convincing positives are bold, with a blue overline. Blue shading indicates residues that are solvent-exposed in the Alphafold-predicted structure. **(D)** Alphafold-predicted surface views (AF-Q9NZU5-F1) of Lmcd1. Dark blue indicates solvent-exposed residues in peptide v17 (residues 309–328); light blue indicates solvent-exposed residues in peptide u9 (residues 113–138). Residue W328 was shaded white for visibility.

**FIGURE 8:**
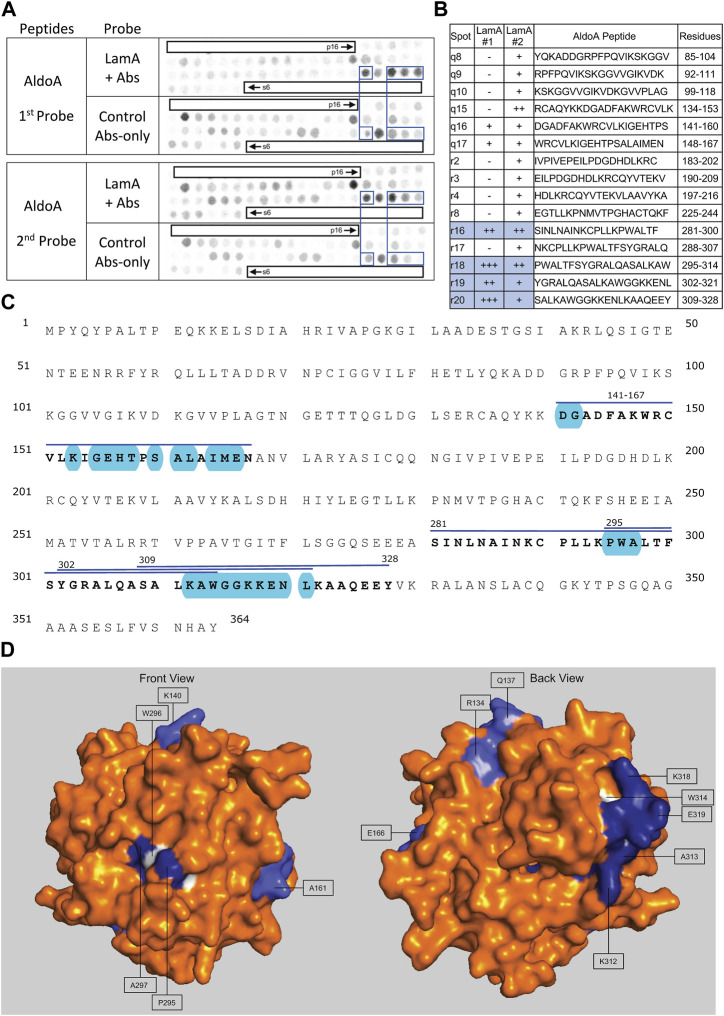
Recombinant lamin A binding to arrayed AldoA peptides. **(A)** Peptide array results from two independent experiments. “first probe” shows two identical arrays, one probed with lamin A protein and detected using primary (lamin A/C) and secondary antibodies, the other (“control”) probed only with detecting antibodies. Paired blue boxes indicate convincing positive spots and corresponding controls. **(B)** Table summarizing peptide array results, listing each AldoA peptide to which lamin A bound weakly (+), moderately (++) or strongly (+++) above background in each experiment, and their amino acid sequence and position in the full-length protein. Convincing positives are shaded blue in the table and indicated by a blue bar in the amino acid sequence. **(C)** Amino acid sequence of human AldoA residues 1–364. Residues in four convincing positives are bold, with a blue overline. Blue shading indicates residues that are solvent exposed in the crystal structure. **(D)** Surface views of the human AldoA crystal structure (PDB: 1ALD). Dark blue indicates solvent-exposed residues in peptides r18–r19 (residues 295–321). Light blue indicates solvent-exposed residues in peptide r16 (residues 281–314). Residues W296 and W314 were shaded white for visibility, because these Trp residues are invariant components of a proposed lamin-binding motif shared with other partners.

**FIGURE 9:**
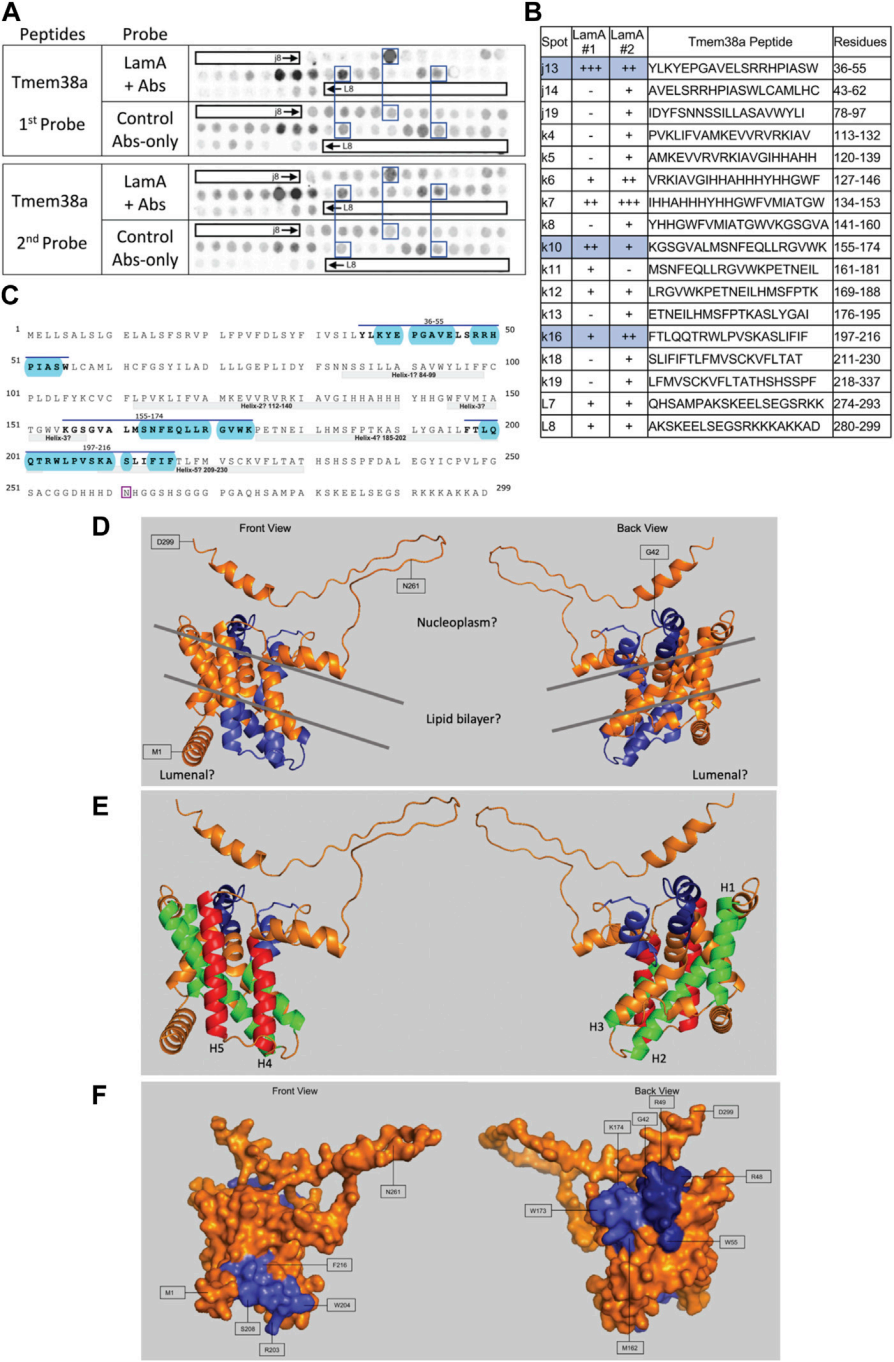
Recombinant lamin A binding to arrayed Tmem38A peptides. **(A)** Peptide array results from two independent experiments. “first probe” shows two identical arrays, one probed with lamin A protein and detected using primary (lamin A/C) and secondary antibodies, the other (“control”) probed only with detecting antibodies. Paired blue boxes indicate convincing positive spots and corresponding controls. **(B)** Table summarizing peptide array results, listing each Tmem38A peptide to which lamin A bound weakly (+), moderately (++) or strongly (+++) above background in each experiment, and their amino acid sequence and position in the full-length protein. Convincing positives are shaded blue in the table and indicated by a blue bar in the amino acid sequence. **(C)** Amino acid sequence of human Tmem38A residues 1–299. Residues in three convincing positives are marked by a blue overline. Blue shading indicates residues that are solvent-exposed in the Alphafold-predicted structure. Residue N261, mutated in Emery-Driefuss muscular dystrophy [“p.N260D”; (Meinke et al., 2020)], is boxed. Light gray bars indicate five bilayer-spanning α-helices predicted by Alphafold, as depicted in panel **(E). (D,E)** Ribbon views and corresponding surface view **(F)** of the Alphafold-predicted structure of human Tmem38A (AF-Q9H6F2-F1). **(D)** Dark blue indicates solvent-exposed residues in the strongest-binding peptide, j13 (residues 36–55). Light blue indicates solvent-exposed residues in convincing peptides k10 (residues 155–174) and k16 (residues 197–216). Gray lines crudely approximate the position of the lipid bilayer. **(E)** Green indicates predicted bilayer-spanning α-helices H1–H3. Red indicates predicted bilayer-spanning α-helices H4 and H5. Dark blue indicates putatively exposed lamin-binding residues 36–55; light blue indicates putatively exposed residues 158–174.

We screened 11 candidates, favoring smaller proteins to fit more per array. Seven candidates are reported here; four will be reported with the brain proteome. Each candidate was displayed as a series of 20-mer synthetic peptides (staggered by seven residues) on cellulose (“peptide array”). The entire array was then probed with purified recombinant mature lamin A “tail” residues 385–646, which includes the flexible linker (residues 385–429) and Ig-fold domain (residues 430–544) shared with lamin C, plus disordered C-terminal residues 545–646 unique to mature lamin A. We used two identical arrays. One array was incubated first with recombinant lamin A, and then with primary (anti-lamin A/C) and secondary antibodies to detect the bound lamins, as shown in panels A of Figures 3–9 (first probe: LamA + Abs). To control for array ‘stickiness’ during their first use, our negative control for the first experiment was to incubate an identical array first (and solely) with antibodies, as shown in panels A of Figures 3–9 (first probe: control Abs-only). For the second full experiment, both arrays were urea-stripped, probed with lamin A protein and antibodies (Figures 3–9, second probe: LamA + Abs), then urea-stripped again and probed with antibodies alone (Figures 3–9, second probe: control Abs-only). The arrays also included peptides representing the lamin A tail itself (residues 385–664), and peptides unique to either lamin C (DEDEDGDDLLHHHHVSGSRR) or “progerin” (CGQPADKASASGSGAQSPQN and ADKASASGSGAQSPQNCSIM), or nuclear membrane protein emerin as positive control. Because many peptides gave high (Ab-only) backgrounds, spot signals were scored independently by two individuals as either weak (+), moderate (++) or strong (+++), compared to the corresponding Ab-only control (panels B of Figures 3–9). Potential sites of Lamin A-binding in each candidate were judged based on signal intensity and consistency (positive in both experiments, or consecutive overlapping peptides), and annotated in the full amino acid sequence (panels C of Figures 3–9). For candidates with known atomic structures, we used PyMol and blue shading to determine if putative lamin-binding peptides were plausibly solvent-exposed. For structurally uncharacterized candidates (Lmcd1, Tmem38a), we relied on Alphafold predictions.

### Peptide array results suggest lamin A tails can interact with neighboring Ig-folds

We included lamin A peptides in the arrays both to query tail-tail interactions, and as a positive control to identify the epitope recognized by the detection system’s lamin A/C antibody. Our detection system (primary anti-lamin A/C and secondary antibodies) was strongly positive for lamin A peptides w16, w17 and x17 (black boxes, Figure 3A, first probe, control Abs-only). The overlapping peptides w16 (aa 464–483; MGNWQIKRQNGDDPLLTYRF) and w17 (aa 471–490; RQNGDDPLLTYRFPPKFTLK) are located in the Ig-fold shared by lamin A and lamin C; from this we deduced the epitope resided in residues 464–490 (gray; Figures 3C,D). Positive x17 (aa 611–630; ISSGSSASSVTVTRSYRSVG) was unique to lamin A, hence ruled out as the primary epitope. We speculate the epitope includes “^480^TYR,” since peptide x17 included homologous residues ‘^625^SYR’. However, this and other background positives remain unexplained.

We detected consistently strongest specific binding of lamin A tails to prelamin A peptide W14 (aa 450–469; KFVRLRNKSNEDQSMGNWQI) and slightly lower signals for overlapping peptides W19 (aa 485–504; PKFTLKAGQVVTIWAAGAGA) and X1 (aa 499–518; AAGAGATHSPPTDLVWKAQN; spots blue-boxed in Figure 3A; scored in Figure 3B). All three positives were located in the Ig-fold, as shown in the amino acid sequence (gray underbar, Figure 3C) and depicted in Figure 3D. The strongest positive, peptide w14, includes many solvent-exposed residues that snake around the bottom and back of the Ig-fold (dark blue in Figure 3D). The other positives, also largely solvent-exposed, occupy most of the “bottom” and “back” surfaces of the Ig-fold (light blue; Figure 3D). These proposed lamin tail-tail interaction regions do not overlap the binding site for BANF1 [pink; front view in Figure 3D; (Samson et al., 2018)]. Lamin A tails did not bind detectably to the lamin C-specific peptide “y3” (Figure 3A) or progerin-specific peptides (data not shown). In the context of lamin filaments, these results suggested that an unidentified region(s) of the lamin A tail (Ig-fold, A-specific disordered region, or both) can interact with the “bottom” and “back” of neighboring Ig-folds (Figure 3D), consistent with an elegant molecular crosslinking study of native lamin A filaments in living cells (Makarov et al., 2019).

### Lamin A binding to emerin

The antibody-only control gave puzzlingly strong recognition of emerin, especially peptides y6 (aa 15–24; LLRRYNIPHGPVVGSTRRLY) and z11-z12 (aa 190–216; FMSSSSSSSSWLTRRAIRPENRAPGAG), and also broadly recognized the N-terminal region (y4–y10), middle (y16–y18; aa 85–118) and C-terminal region (z14–z18; aa 211–254) (Figures 4A, B; first probe, control Abs-only). We first checked, and ruled out, potential switching of the two images. Against this formidable and disappointing background, one peptide was weakly positive in both experiments: emerin z10 (aa 183–202; PTSSSTSFMSSSSSSSSWLT). This peptide resides in one of two large fragments of emerin, namely residues 1–132, and 159–220— each sufficient to bind lamin A (Berk et al., 2014). This result suggested lamin A contacts emerin residues 183–202 near the transmembrane domain, between two peptides (SAYQS region and R-peptide) involved in emerin-emerin association (Berk et al., 2014; Figure 4C).

### Robust binding of lamin A tails to Perm1

We detected strong lamin A binding to three Perm1 peptides (Figures 5A, B), namely h5 (aa 441–460; SSGLVSTPVPRADAAGLAWP), i4 (aa 593–612; EENEEAEAAAAGQDPAGVQW) and j2 (aa 737–756; FVAFATWAVRTSDPHTPDAW), and consistent weaker binding to h16 (aa 529–548; QTATGAHGGPGAWEAVAVGP), as annotated in the full amino acid sequence of Perm1 (Figure 5C). We were unable to evaluate these peptides in a structural context, since Perm1 is almost entirely disordered, except to note that the proposed lamin A-binding peptides are all located in the C-terminal half of Perm1, and that two sites have predicted α-helicity (Figure 5D). Our conclusion that Perm1 can bind mature lamin A was unexpected, since a previous yeast two-hybrid study reported detectable binding of Perm1 (known as C1orf179) only to progerin (Dittmer et al., 2014).

### Weak lamin A tail binding to the N-terminal region of Csrp3 (muscle LIM protein “MLP”)

Csrp3, also known as “muscle LIM protein” (MLP), is a nucleocytoplasmic shuttling protein that crosslinks and bundles F-actin and influences myocyte remodeling and responses to cardiac hypertrophy (Hoffmann et al., 2014). We observed weak lamin A tail binding to Csrp3 peptide s7 (aa 1–20; MPNWGGGAKCGACEKTVYHA) in both experiments, and weak binding to overlapping peptides s9–s11 (aa 15–48; KTVYHAEEIQCNGRSFHKTCFHCMACRKALDSTT) and t5–t6 (aa 127–146; VYAAEKVMGGGKPWHKTCFRCAICGKS) in the second experiment (Figures 6A, B). We were unconfident about the strong signals for peptides s14–s15 in the second experiment due to their high background signals in the first experiment (Figures 6A, B). The full amino acid sequence of Crsp3 is shown in Figure 6C. Csrp3 has two zinc-binding domains: LIM domain-1 (residues 7–66; Figure 6D) mediates Csrp3 dimerization, whereas LIM domain-2 (residues 119–176; Figure 6D, right) binds F-actin (Hoffmann et al., 2014). Our array data suggests lamin A binds disordered residues 1–6 and solvent-exposed residues 7–20 of LIM domain-1 (shaded blue in Figure 6D) and might therefore influence Csrp3 dimerization.

### Strong lamin A binding to Lmcd1 (LIM and cysteine-rich domain 1)

We detected strong consistent lamin A tail binding to Lmcd1 peptide v17 (aa 309–328; SGCDEIIFAEDYQRVEDLAW), and consistent but weaker binding to three peptides (u8–u10) that shared residues 113–132 (FDTITYEWAPPGVTQKLGLQ) (Figures 7A, B). Given the limitations of this assay we drew no conclusions from the faint signals seen with peptide t13 (aa 1–20), and other peptides that were positive only in the second experiment (Figures 7A, B). Convincing positives were annotated in the amino acid sequence of Lmcd1 (Figure 7C). Lmcd1 has a putative protein-protein interaction (“PET”) domain (residues 99–206), a disordered region (residues 200–235) and two predicted LIM zinc-binding domains (residues 241–306, and 307–365; Figure 7C). The strongest lamin A-binding site (peptide v17, in the second zinc-binding domain) is almost entirely solvent-exposed in the Alphafold-predicted structure of Lmcd1 (shaded blue in Figure 7D). The weaker lamin A-binding site (residues 113–132) in the putative protein-protein interaction region includes eight predictedly solvent-exposed residues (shaded blue in Figure 7D).

### Strong binding of lamin A to AldoA

Lamin A tails showed consistently strong binding to overlapping AldoA peptides r16 (aa 281–300; SINLNAINKCPLLKPLLKPWALTF), r18 (aa 295–314; PWALTFSYGRALQASALKAW), r19 (aa 302–321; YGRALQASALKAWGGKKENL) and r20 (aa 309–328; SALKAWGGKKENLKAAQEEY; Figures 8A, B). We were intrigued to see that peptide r16 (aa 281–314) was mostly buried in the X-ray crystal structure (Figure 8D). The exceptions were residues P295, W296, A297 (“PWA”), on a concave surface (shaded blue in “front” view, Figure 8D), and residues K312, A313 and W314 (‘KAW’; shaded blue or white in Figures 8C, D). “PWA” was shared by overlapping AldoA peptides r16 and r18 (Figure 8B), and “KAW” by three strongly positive overlapping peptides (r18–r20; Figure 8B). We noticed similar “AW” residues in strongly positive peptides from Lmcd1 (^326^LAW; Figure 7B) and Perm1 (^457^LAW, ^539^GAW, ^754^DAW; Figure 5B); comparison suggested elements of a potential shared motif (see *Discussion*).

### Robust lamin A tail binding to nuclear membrane protein Tmem38a

Lamin A showed consistently strong binding to Tmem38A peptide j13 (aa 36–55; YLKYEPGAVELSRRHPIASW; Figures 9A, B). Other consistent positives were k10 (aa 155–174; KGSGVALMSNFEQLLRGVWK) and k16 (aa 197–216; FTLQQTRWLPVSKASLIFIF). We disregarded weaker positives k12 (aa 169–188; LRGVWKPETNEILHMSFPTK) and L7–L8 (aa 274–299; QHSAMPAKSKEELSEGSRKKKAKKAD). We disregarded strong peptides k6–k7 because they were inexplicably positive in the antibody-only control (Figure 9A, first probe). Tmem38 is an integral membrane protein that localizes at the nuclear envelope inner membrane in muscle cells (Robson et al., 2016). Tmem38A is homologous to trimeric intracellular cation type-A (TRIC-A) channels, crystal structures for which were determined only in procaryotic and *C. elegans* orthologs (Kasuya et al., 2016). The structure of human Tmem38A has not been determined and was variously predicted to have three (Zhou et al., 2014) or four (Meinke et al., 2020) transmembrane helices. Our Alphafold prediction suggests five membrane-spanning helices (gray bars in Figure 9C; helices 1–3 colored green and helices 4–5 red in Figure 9E), three membrane-adjacent helices (residues 1–18, 236–249, and 285–295), and at least one “kinked” (partially bilayer-inserted) helix. Putative helix-2 and helix-5 are quite long and probably protrude beyond the lipid bilayer. Since Alphafold does not depict the lipid bilayer, we approximated crudely as depicted by gray lines in Figure 9D. This Alphafold-predicted topology suggests the C-terminus, lamin A-binding peptide j13 (residues 36–55) and most of lamin-binding peptide k10 (residues 158–174) are exposed and accessible *in vivo* (AF-Q9HF2-F1; residues 36–55 dark blue, residues 158–174 light blue, in Figures 9D–F). The third identified site (peptide k16) is predicted to localize in the lumen (Figure 9D), inaccessible to lamins *in vivo*.

## Discussion

This study identified lamin A/C-associated proteomes from two native tissues, heart and skeletal muscle, often perturbed in laminopathy and frailty. We begin with the limitations of this study. First, we recovered fewer-than-expected known partners and few integral membrane proteins from heart and skeletal muscle. We attribute this deficit to the challenges inherent in solubilizing lamin-associated proteins from relatively insoluble nuclear lamina networks in cells filled with insoluble contractile networks, because our same solubilization protocol yielded numerous known partners and integral membrane proteins when applied to softer tissue (brain; manuscript in preparation). Thus, different (e.g., proximity labeling) strategies could still be fruitfully applied in striated tissues. Second, with respect to the ∼51 proteins that showed differential lamin A/C association in frail (IL10-KO) tissue, we did not determine *why* association changed. Changes could be due to many factors downstream of chronic inflammation, including gross changes in the abundance, posttranslational modification or nuclear localization of any given protein. A subset of proteins identified with high-confidence by mass spectrometry had large magnitude (e.g., >200-fold), yet statistically insignificant, changes in lamin A/C association in IL10-KO tissue, as shown in Supplementary Table S5. This we attributed to the small sample size of this study, or more speculatively to posttranslational modifications that ‘removed’ specific peptides from the analysis. Nor did we stain candidates by indirect immunofluorescence to visually confirm lamin A/C association or changes thereof in IL10-KO versus control tissues. Finally, to minimize false positives, we probed the peptide arrays stringently, with lamins at 190 nM concentration, within the range of most but not all affinities measured for emerin [e.g., 40 nM for lamin A; 4–500 nM for other partners; see Berk et al. (2013)]. In cells, lamin concentrations near the inner nuclear membrane are estimated at >10 uM (Holaska et al., 2003).

Despite these limitations, our major conclusion is that these native lamin A/C proteomes from heart and skeletal muscle are usefully enriched in novel partners relevant to laminopathy and frailty. Among the seven candidates screened here for lamin A-binding, two (Fabp4, Gins3) gave negative results, one was detectably positive (Csrp3) and four (Lmcd1, AldoA, Perm1, Tmem38A) showed strong binding that allowed us to map and evaluate putative lamin A-binding sites at the molecular level. WebLogo analysis of strong-binding peptides showed patterns of frequent residues around an invariant Trp (Supplementary Figure S2), which is barely visible in Lmcd1 and AldoA (white in Figure 7C, Figure 8C). Further analysis of these and additional lamin-binding peptides will be needed to determine if it is possible to define a lamin-binding motif, or locate its hypothetical docking site on lamin A.

### Independent evidence for molecular interplay between lamin A/C tails

Our evidence suggests lamin A “tails” (including the Ig-fold), which extend flexibly away from the filament backbone, have the capacity to interact with nearby tails either on the same filament, or possibly different filaments. These results independently support previous crosslinking evidence that lamin tails can associate with each other in the context of native filament networks (Makarov et al., 2019). Furthermore our lamin-binding peptides were located on the “bottom” and “back” of the Ig-fold, away from the binding site for BANF1 (Samson et al., 2018), suggesting these proposed tail-tail interactions would not interfere with BANF1, an essential partner. More work is needed to determine which region of lamin A (Ig-fold, or disordered A-specific tail) contacts neighboring Ig-folds, and whether such interactions affect filament dynamics *in vivo* (Makarov et al., 2019).

### CSRP3, LMCD1, ALDOA, PERM1: candidate genes for Emery-Dreifuss muscular dystrophy that may also provide insight into frailty-related muscle weakness?

The genes encoding four novel lamin A-binding proteins identified in this study are worth testing for potential genetic linkage to “unmapped” EDMD patients. These proteins may also be relevant to understanding the pervasive muscle weakness phenotypes of frail patients.

#### Csrp3

Csrp3 was lamin A/C-associated in both heart and muscle and directly binds lamin A, yet was unaffected by IL10-KO. CSRP3 is a plausible EDMD candidate gene because Csrp3 is a mechanosensitive transcription regulator in muscle and cofactor for MyoD1 (Mathiesen et al., 2019), and also binds LC3 and promotes autophagy as a mechanism of protection against muscular dystrophy (Cui et al., 2020). Autophagy is an important protective mechanism (Choi and Worman, 2013). Mitochondrial autophagy is deficient in IL10-KO skeletal muscle (Ko et al., 2016). Two other LIM domain proteins involved in autophagy were also identified in our study: Fhl1 in heart and muscle (Sabatelli et al., 2014) and Fhl2 in heart (Xia et al., 2017). Peptide mapping showed lamin A binds one of two LIM domains in Csrp3, and one of two LIM domains in Lmcd1. The significance of lamin A binding to LIM domains, *per se*, is unknown, since LIM domains are widespread and diverse mediators of protein-protein interactions (Kadrmas and Beckerle, 2004). We also identified Lmo7 (LIM domain only 7) in the heart proteome; Lmo7, a signaling transcription factor genetically linked to Emery-Dreifuss muscular dystrophy, binds emerin (Holaska et al., 2006) but to our knowledge is untested for binding to lamin A.

#### Lmcd1

Lmcd1 association with lamin A/C was reduced significantly in frail (IL10-KO) muscle. Lmcd1 is a positive regulator of muscle mass and muscle fiber size, and battles fatigue by repressing myoregulin (Ferreira et al., 2019). Lmcd1 is a Z-disc protein that responds to mechanical load (Luosujärvi et al., 2010), mediates cardiac hypertrophy (Frank et al., 2010), and is a shuttling transcription repressor that blocks the DNA-binding activity of transcription activator GATA6, which regulates lung and cardiac tissue-specific promoters (Rath et al., 2005). Lamin A and GATA6 both interact with the second LIM-domain of Lmcd1, suggesting lamin A might compete with GATA6 and potentially free GATA6 to promote transcription. Lmcd1 is functionally relevant to EDMD. Its reduced association with lamin A/C in IL10-KO muscle suggests Lmcd1 is also relevant to sarcopenia and fatigue in frailty and warrants further testing.

#### AldoA

Novel lamin A-binding protein glycolytic enzyme AldoA, identified in both heart and muscle, was significantly reduced in IL10-KO hearts. AldoA links glycolysis to ribosome biogenesis (Schwarz et al., 2022), and has protective roles in the heart related to Notch signaling (Luo et al., 2021). Our results are consistent with AldoA functioning in the nucleus and further suggest AldoA is relevant to the mechanisms of laminopathy and frailty. Further studies of AldoA are clearly warranted.

#### Perm1

Transcription co-activator Perm1, an essential regulator of mitochondrial and cardiac energetics (Oka et al., 2022) and fatty acid metabolism (Huang et al., 2022), was lamin A/C-associated in hearts and recognized by mature lamin A in the peptide array. Perm1 is attractive to consider in the context of frailty because it also regulates genes required for endurance exercise and promotes mitochondrial biogenesis and oxidative capacity in muscle (Cho et al., 2016; Cho et al., 2019; Cho et al., 2021). As a disordered protein, Perm1 lacks conventional structure and is likely controlled by differential posttranslational modifications. Other partners for Perm1 in the heart include transcription co-activators BAG6, Kank2, ERRα and PGC-1α (Oka et al., 2022); among these we identified Kank2 as lamin A/C-associated (and unaffected by IL10-KO) in the heart.

Calpain-1 catalytic subunit, a mitochondrial protein not known to enter the nucleus, was significantly reduced in IL10-KO skeletal muscle (*p* < 0.0032), as were many other mitochondrial proteins. Reduced mitochondrial proteins (even as contaminants) was expected, since mitochondrial loss is a phenotype of the IL10-KO model (Ko et al., 2016). Interestingly, we also identified legitimate regulators of mitochondrial biogenesis associated with lamin A/C. Perm1, a key positive regulator of mitochondrial biogenesis, was unaffected in IL10-KO muscle. Fbp2, a key negative regulator that represses nuclear-encoded mitochondrial genes (Huangyang et al., 2020), showed significantly reduced lamin A/C association in IL10-KO muscle (*p* < 0.021). Further studies are needed to test the biological relevance and subnuclear localizations of Perm1 and Fbp2, because one cannot predict if a given partner is active or sequestered (inactive) when bound to lamins. Furthermore, lamins are not solely associated with silent chromatin; subpopulations of lamin A and lamin C localize in the nuclear interior and support transcriptional activity (Naetar et al., 2017). We speculate that if Fbp2 were normally sequestered by association with lamins, then reduced association in IL10-KO muscle would imply that Fbp2 gains freedom to repress mitochondrial genes.

### Lamin A-binding sites in Tmem38 did not include residue N261, mutation of which causes EDMD

Tmem38A has two unreconciled roles: on one hand, an intracellular cation channel at the nuclear inner membrane; on the other, a muscle-specific regulator of 3D chromosome organization (Czapiewski et al., 2016; Robson et al., 2016). Given the current uncertainty about the structure and topology of Tmem38a, our best prediction is that two of our three identified lamin-binding sites in Tmem38a are exposed and accessible to lamins *in vivo* (Figures 9D, E). Interestingly, none of our lamin A-binding peptides included residue N261 (boxed in Figure 9C), the site of a recently-reported EDMD-causing mutation, p.N260D [numbered without the initiating Met; (Meinke et al., 2020)]. Since residue N261 is predicted to be exposed (Figure 9D), we speculate that this mutation disrupts Tmem38A in some other way, for example by perturbing Tmem38A binding to a different EDMD-relevant partner such as emerin, Fhl1, Lemd3/Man1, Sun1, Sun2, Tmem201, Tmem43/Luma, Nesprin-1, Nesprin-2 or Nesprin-3 (Meinke et al., 2020). Further work on the structure and function of Tmem38a is needed to understand this fascinating and disease-relevant protein.

### Fabp4 and Gins3: significantly higher lamin A/C association in IL10-KO muscle and heart, respectively, but no detectable binding to lamin A

Lamin A gave no detectable binding to Fabp4 or Gins3 peptides (data not shown). Since negative results are inconclusive, further studies with full-length proteins will be needed to retest potential binding to lamin A, and also test lamin C. One enduring mystery is how lamins A and C, with residues 1–566 identical, nevertheless form separate filaments (Shimi et al., 2015) and have distinct roles in metabolism and lifespan (Fong et al., 2006; Lopez-Mejia et al., 2014) and tissue-specific 3D chromosome organization (Wong et al., 2021b). As lamin A/C-associated and IL10-KO-affected candidates important for metabolism (Garin-Shkolnik et al., 2014; Das et al., 2015; Hotamisligil and Bernlohr, 2015; Liu et al., 2022), Fabp4 and Gins3 both warrant further investigation.

## Data Availability

The raw mass spectrometry data for this study are publicly available via figshare for heart (10.6084/m9.figshare.24187089) and skeletal muscle (10.6084/m9.figshare.24187086).

## Supplementary material

SUPPLEMENTARY FIGURE S1 Mice used in this study. (Top) Western blots of lamin A/C immunoprecipitates from the hearts of all 19 starting mice, collected on the same day. To normalize against blot-to-blot variation (especially when detecting *O*-GlcNAc) each gel included samples from both IL10-KO and control hearts. The number above each lane (e.g., “1, 2”) corresponds to the mouse listed in the Table (e.g., “M001”, “M002”). SDS-resolved samples were transferred and probed first for the *O*-GlcNAc modification (left; α-*O*-GlcNAc), then stripped and re-probed for lamin A/C (α-lamin A/C) using an antibody different from the immunoprecipitating antibody. The blots shown are representative of two independent replicates. We tested *O*-GlcNAc to ask if lamin A was hyper-*O*-GlcNAcylated in frailty. Because posttranslational modifications on native lamin A can shift its migration in gels, lamin A signals were collected as indicated by the bracket at right. *O*-GlcNAc-to-lamin A ratios trended higher in IL10-KO hearts, but we considered a potential difference insignificant given the variation between mice and our inability to rule out *O*-GlcNAcylation of co-migrating proteins. (Bottom) Table that details genotype, age, weights and notes (during tissue collection) about the 19 mice considered for this study, and shows which mouse samples were selected for proteomic analysis. IL10-KO genotype denoted as “IL10^tm^” in the Table.

SUPPLEMENTARY FIGURE S2 Clustal Omega and WebLogo display of amino acids common to lamin-binding peptides near an invariant Trp residue.

SUPPLEMENTARY TABLE S1 Unfiltered mass spec results for heart and muscle (WT vs IL10-KO, denoted as “IL10^tm^” or “KO”).

SUPPLEMENTARY TABLE S2 Proteins identified in heart and muscle (WT vs IL10-KO, denoted as “IL10^tm^” or “KO”) after normalization to lamin A (‘prelamin A’) in each sample.

SUPPLEMENTARY TABLE S3 Proteins identified in heart and muscle (WT vs IL10-KO, denoted as “IL10^tm^” or “KO”) after both normalization to lamin A, and filtering to remove most mitochondrial proteins and all ribosomal proteins and keratin as presumed contaminants.

SUPPLEMENTARY TABLE S4 Same as Supplementary Table S3, with protein names matched with gene names and manually curated as described in Methods.
