# Supplementary figures and images for: Native lamin A/C proteomes and novel partners from heart and skeletal muscle in a mouse chronic inflammation model of human frailty
Source: Front Cell Dev Biol. 2023 Oct 23;11:1240285. doi: 10.3389/fcell.2023.1240285 (PMC10626543; doi:10.3389/fcell.2023.1240285)

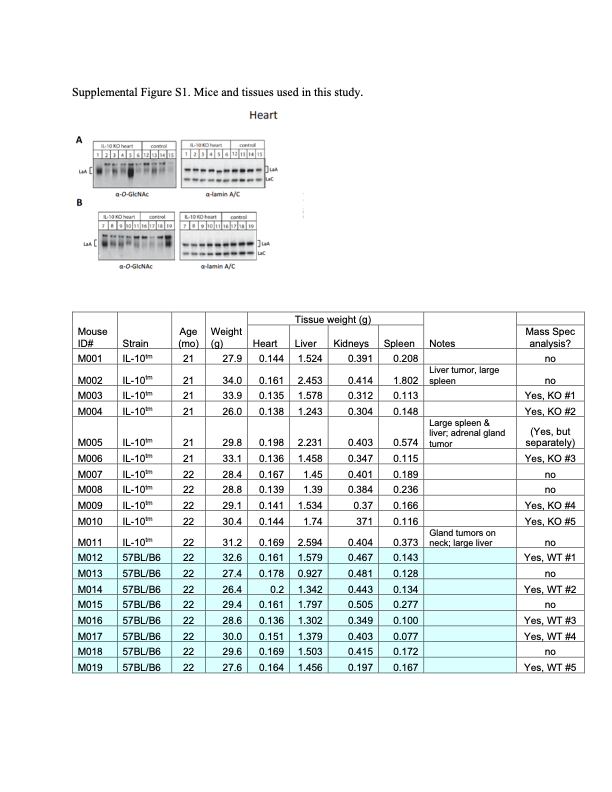

Supplement: Supplementary file 2 [file Image1.TIFF]

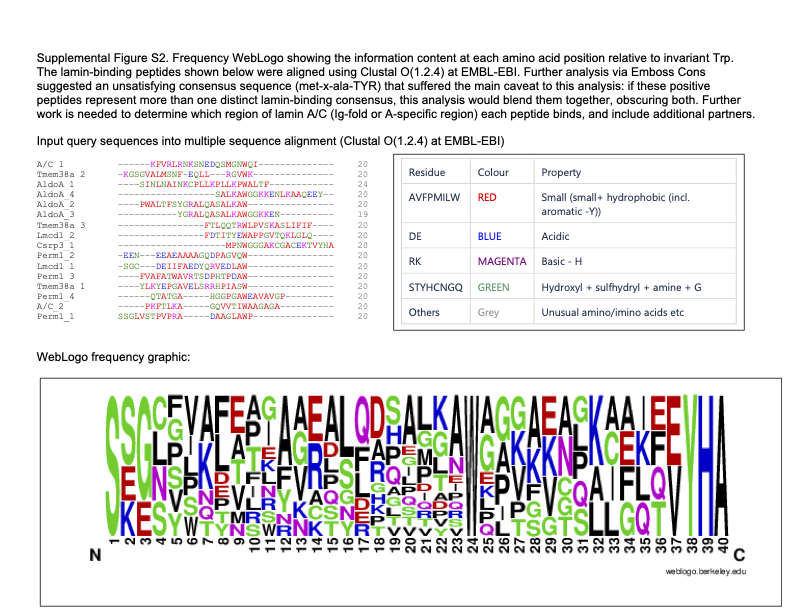

Supplement: Supplementary file 6 [file Image2.TIFF]
